# Impact of the COVID-19 Lockdown on Air Quality and Resulting Public Health Benefits in the Mexico City Metropolitan Area

**DOI:** 10.3389/fpubh.2021.642630

**Published:** 2021-03-25

**Authors:** Iván Y. Hernández-Paniagua, S. Ivvan Valdez, Victor Almanza, Claudia Rivera-Cárdenas, Michel Grutter, Wolfgang Stremme, Agustín García-Reynoso, Luis Gerardo Ruiz-Suárez

**Affiliations:** ^1^Centro de Ciencias de la Atmósfera, Universidad Nacional Autónoma de México, Ciudad de Mexico, Mexico; ^2^CONACYT, Centro de Investigación en Ciencias de Información Geoespacial, Querétaro, Mexico; ^3^Instituto Nacional de Ecología y Cambio Climático, Coordinación de Contaminación y Salud Ambiental, Ciudad de Mexico, Mexico

**Keywords:** COVID-19, Fourier series, meteorology, remote sensing, air pollutant emissions

## Abstract

Meteorology and long-term trends in air pollutant concentrations may obscure the results from short-term policies implemented to improve air quality. This study presents changes in CO, NO_2_, O_3_, SO_2_, PM_10_, and PM_2.5_ based on their anomalies during the COVID-19 partial (Phase 2) and total (Phase 3) lockdowns in Mexico City (MCMA). To minimise the impact of the air pollutant long-term trends, pollutant anomalies were calculated using as baseline truncated Fourier series, fitted with data from 2016 to 2019, and then compared with those from the lockdown. Additionally, days with stagnant conditions and heavy rain were excluded to reduce the impact of extreme weather changes. Satellite observations for NO_2_ and CO were used to contrast the ground-based derived results. During the lockdown Phase 2, only NO_2_ exhibited significant decreases (*p* < 0.05) of between 10 and 23% due to reductions in motor vehicle emissions. By contrast, O_3_ increased (*p* < 0.05) between 16 and 40% at the same sites where NO_2_ decreased. During Phase 3, significant decreases (*p* < 0.05) were observed for NO_2_ (43%), PM_10_ (20%), and PM_2.5_ (32%) in response to the total lockdown. Although O_3_ concentrations were lower in Phase 3 than during Phase 2, those did not decrease (*p* < 0.05) from the baseline at any site despite the total lockdown. SO_2_ decreased only during Phase 3 in a near-road environment. Satellite observations confirmed that NO_2_ decreased and CO stabilised during the total lockdown. Air pollutant changes during the lockdown could be overestimated between 2 and 10-fold without accounting for the influences of meteorology and long-term trends in pollutant concentrations. Air quality improved significantly during the lockdown driven by reduced NO_2_ and PM_2.5_ emissions despite increases in O_3_, resulting in health benefits for the MCMA population. A health assessment conducted suggested that around 588 deaths related to air pollution exposure were averted during the lockdown. Our results show that to reduce O_3_ within the MCMA, policies must focus on reducing VOCs emissions from non-mobile sources. The measures implemented during the COVID-19 lockdowns provide valuable information to reduce air pollution through a range of abatement strategies for emissions other than from motor vehicles.

## Introduction

The coronavirus disease (COVID-19) spread rapidly around the world in early 2020, changing anthropogenic activities permanently (1). The first cases of COVID-19 in Mexico were diagnosed on 27 February 2020 (2). To control the COVID-19 outbreak in Mexico, social distancing measures were implemented following a scheme of 2 lockdown phases. Unofficially, lectures and student attendance to schools were the first activities suspended on 17 March, while all academic activities were suspended completely on 24 March after exceeding one thousand COVID-19 confirmed cases in the whole country (Supplementary Figure 1) (3), which led the Federal Government to declare the COVID-19 Phase 2. Followed by Phase 2, on 30 March, partial lockdown measures targeting non-essential activities were introduced for shopping, leisure, public administration services and public gatherings of more than 25 people (Table 1) (4). Finally, with the declaration of COVID-19 Phase 3 on 21 April, the total lockdown prohibited all non-essential activities remaining in action (5). In particular, within the Mexico City Metropolitan Area (MCMA), the total lockdown measures included the suspension of all non-essential industrial production and supplies trading, reduction of the public transport service and the enforcement of “a day without a car” program for all petrol vehicles (Supplementary Figure 1; Table 1).

**Table 1:** Summary of the COVID-19 outbreak propagation and lockdown measures implemented within the MCMA.

**Date**	**Lockdown**	**COVID-19 outbreak**	**Measures**
28/02/2020	–	First positive cases detected.	Normal social and economic activities. Use of face masks and hand sanitizer gel recommended.
17/03/2020	Included in Phase 2 in this study.	Accelerated increase of positive cases.	Unofficial suspension of school attendance. Suspension of sports events and concerts.
24/03/2020	Phase 2	Exceedance of 1,000 positive cases.	Official suspension of all academic and school activities.
30/03/2020			Social distancing measures are mandatory. Reduction of public transport services. Prohibition of meetings of >25 people. Ceasing of services in malls, public parks and museums, sports and leisure facilities, bars, nightclubs, and religious facilities.
21/04/2020	Phase 3	Community transmission of COVID-19	Mandatory use of face masks and hand sanitizer gel. Enforcement of the program a day without car. Closing of some metro stations and increase of public buses frequency service operating a <50% of capacity. Suspension of local and federal government services. Ceasing of operations at all non-essential industries.

Numerous studies focusing on evaluating the urban air quality during the COVID-19 lockdown have documented significant reductions in air pollutants concentrations worldwide, associated with reduced emissions from anthropogenic activities, mainly from reduced vehicle activity (6–10) (Supplementary Table 1). The fact that lockdowns improve air quality in urban environments as a result of restrictions targeting reducing anthropogenic activities is expected when compared with prior business as usual periods. Nevertheless, the degree of air pollutants reduction may vary from city-to-city due to local factors such as the severity of lockdown measures, distribution of emission sources, meteorology and trends in pollutant emissions, complicating the precise quantification of changes in air pollutants concentrations, and their possible public health benefits during the COVID-19 lockdown (11, 12). While most studies have focused on comparing pre-lockdown air pollutant concentrations with those under lockdown, the impact of meteorology, and long-term trends on the latter has received less consideration.

The COVID-19 lockdown can be seen as an unplanned experiment to study the effect on air quality of extraordinary reductions in anthropogenic activities. Furthermore, this lockdown may help to identify an air quality baseline to achieve in non-lockdown conditions. Nowadays, most existing public policies aiming to reduce air pollution are focused on the abatement of long-term emissions and increasing energy efficiency standards. Therefore, minimising the influences of meteorology and long-term policies on air pollutant concentrations is required to precisely detect changes in their concentrations ascribed to new interventions (13–15). An even more challenging task is to extract from observations the signal of short-term interventions to reduce extremely high pollution levels since these can be obscured by meteorological fluctuations (16). For instance, air pollutants may accumulate due to air stagnation related to meteorological conditions favouring dry and stable regimes characterised by a lack of ventilation, presence of temperature inversions, and high-pressure systems with influence at synoptic scale (17, 18). Therefore, a scenario in which changes in air pollutant concentrations during the COVID-19 lockdown could be driven by meteorology rather than by reductions in their emissions must be considered.

With a population of more than 21 million people in the MCMA (19), it can be hypothesised that the unprecedented lockdown measures aiming to control the COVID-19 outbreak resulted in significant reductions in air pollutant concentrations over this period. Such reductions were presumably larger in industrial-vehicle environments than in urban background conditions. This study presents changes in CO, NO_2_, O_3_, SO_2_, PM_10_, and PM_2.5_ (criteria pollutants) during the COVID-19 lockdown in the MCMA. By contrast with most existing studies, the focus of this study was to (i) minimise the influence of seasonality, long-term trends and rapid weather changes on air pollutant concentrations, (ii) observe differences in pollutant concentrations for particular environments associated with improved air quality during the lockdown, and (iii) calculate public health benefits under lockdown conditions. Observations from the MCMA Air Quality Monitoring Network were obtained from representative sites to capture air pollutants behaviour before the lockdown using Fourier series modelling. Hypothesis tests were used to confirm that our models, integrated by constant deviation, long-term trend and seasonal component, fit better the data than a constant value. Predictions of air pollutants from calibrated models were used to calculate anomalies for the lockdown, and these compared to anomalies during the corresponding periods between 2016 and 2019 (baseline). A 4-year baseline was selected to compare the air pollutant concentrations during the lockdown in order to reduce the impact of inter-annual variability on air pollutant levels.

To more precisely quantify the impact of the two lockdown phases on criteria air pollutants within the MCMA, the obtained anomalies were analysed through statistical tests of probability density functions, diurnal cycles, and overall anomalies. Remote sensing observations were also used to confirm our results. Finally, an air quality health index and changes in the health burden associated to potential outdoor pollution exposure were calculated to identify potential health benefits from reduced air pollution during the lockdown. The methodology reported here can be applied in other cities where air pollutants exhibit significant seasonality, long-term trends or are severely affected by meteorology to better quantify the impact of pollution control strategies on short-term scales.

## Materials and Methods

### Air Pollutants Data

Criteria air pollutants have been measured continuously within the MCMA since 1986 by the Atmospheric Monitoring System (SIMAT) of the Mexico City government, and are publicly available as 1-h averages after proper validation using US Environmental Protection Agency standards (20). Air pollutants data recorded during lockdown Phases 2 and 3, and corresponding periods from 2016 to 2019, at monitoring sites representative of traffic (TRA), industrial (IND), commercial (COM), residential (RES), and urban background [upwind [UBN] and downwind [UBS]] sites were downloaded from the SIMAT website (Supplementary Table 2) (http://www.aire.cdmx.gob.mx). Henceforth the lockdown phases are referred to only as Phases 2–3. All selected sites have a minimum data capture of 75% 1-h averages for all air pollutants during the studied periods. Supplementary Figure 2 shows the selected monitoring sites location within the MCMA. Additional details of the monitoring sites location, description and characteristics can be found elsewhere (20).

### Calculation of Anomalies in Air Pollutant Concentrations

To minimise the influence of seasonality and long-term trends, we used an approach based on anomalies in air pollutant concentrations during the lockdown in comparison with a 4-year baseline. The air pollutant anomalies during lockdowns were obtained by subtracting predicted data using truncated Fourier series from observed data as follows. Firstly, a modelled fitted function was computed for all air pollutants using truncated Fourier series with a frequency of *k* = 2 as shown in Equation (1):

(1) $$\begin{array}{l} P\left(t\right)=c_{0}+c_{1}t+\overset{K}{\underset{k=1}{\sum }}\left[a_{k}\text{ cos }\left(2\pi tk\right)+b_{k}\text{ sin }\left(2\pi tk\right)\right] \end{array}$$

where *t* is the normalised time, that is to say, the number of days in a given calendar year divided by 365 to fit a full sine/cosine cycle. The model captures the long-term trend component with the first two terms (*c*_0_ + *c*_1_), while the seasonal component is captured by the aggregation of all terms. The Fourier model is a natural form of modelling seasonal trends, it should be noted that a large number of frequencies could lead to overfitting, therefore, we use only two frequencies for achieving a statistically significant model that visually fits the air pollutants trends. This reduces the possibility of meteorological and seasonal bias in contrast with existing studies (10, 21). The Fourier model was calibrated using daily averages from 2016 to 2019 for all air pollutants to reduce high-frequency variability. For the lockdown periods, the modelled pollutants data were obtained from the model predictions, using *P*(*t*) with the corresponding constants *c*_0_*, c*_1_*, a*_1_*, a*_2_*, b*_1_, and *b*_2_. Finally, the hourly anomalies were calculated as shown in Equation (2):

(2) $$\begin{array}{l} A\left(t\right)=D\left(t\right)-P\left(t\right) \end{array}$$

where *A(t)* are the hourly anomalies, *D(t)* are the hourly observations and *P(t)* are the modelled pollutant daily data for the day *t*. Air pollutants modelling was made using the R software (22).

### Meteorological Analyses

To discard the effects of air pollutants accumulation, days with stagnant conditions in which meteorology might have a significant influence were excluded from the analysis. Stagnant events for the lockdown and baseline periods were obtained using the air stagnation index (ASI), which is a binary index based on daily precipitation thresholds together with surface and upper wind speed (WS) (13, 14). The following daily mean thresholds must be met together to define a day as stagnant: surface WS <3.2 m s^−1^, WS at 500 mb <13 m s^−1^, and daily total precipitation <1 mm. The purpose of calculating the ASI in this work was to discard days with significant meteorological influence on air pollutant concentrations (18), rather than suggesting a cause-effect relationship, an issue that is beyond the scope of this study. The effect of heavy rain on air pollutant concentrations was minimised also by excluding days with heavy precipitation from the analysis (>10 mm as daily average) (23). The removed days account for <30% of the studied period in each year. Hourly surface meteorological data for temperature (T), relative humidity (RH), WS and wind direction (WD) were obtained from the SIMAT website. Data of WS at 500 mb were obtained from the upper-air soundings of the Mexico City International Airport and are readily available at the University of Wyoming data repository (http://weather.uwyo.edu/upperair/sounding.html). Daily precipitation data for the area encompassing the MCMA were obtained from the Level 3 product of the Global Precipitation Measurement (GPM) at 10 km resolution (24). The GPM is an international network of satellites that provide the next-generation global observations of rain and snow.

### Satellite-Based Observations

Changes in surface NO_2_ and CO during lockdown phases 2–3 were compared with those determined in the corresponding period of previous years using satellite-based observations. As for surface observations, days with stagnant conditions and heavy rain were excluded (see section Meteorological Analyses) from the NO_2_ and CO satellite-based analyses. For NO_2_, we used the standard product V003 (25) from the Ozone Monitoring Instrument (OMI) onboard the Aura satellite of the National Aeronautics and Space Administration (NASA), downloaded from the Earth Data Website (https://earthdata.nasa.gov/). From the OMI NO_2_ data product (26), the tropospheric column was used and filtered with a cloud fraction lower than 20% and quality flag 0. NO_2_ distribution maps were constructed using data averaged for the 2016–2019 lockdown corresponding period and compared with 2020 data. Specific details of the methodology for the construction of maps can be found in (27).

The averaged distribution of the CO column over the MCMA during the lockdown phases was reconstructed from the TROPOMI satellite instrument. The TROPOspheric Monitoring Instrument is, as in Nadir geometry, a measuring instrument on-board the Sentinel 5P satellite, which has a sun synchronised orbit and a daily equatorial crossing time at 13:30 LST and a similar overflight time for the MCMA. The swath of the instrument provides daily coverage over the MCMA and data from the level 2 product were downloaded (28, 29). The distribution maps were reconstructed using the oversampling method reported in UNIATMOS (30) with a defined mesh of 50 × 50 grid points. The calculated CO concentration at each grid point represents the average of all measurements with a pixel centre closer than 5 km to each grid point. Five kilometre was empirically determined as a trade off between the mesh grid (ca. 2 km), the noise and density of the data for this period, the footprint of the TROPOMI-measurement and the inhomogeneity expected for the MCMA.

### Mobility Analyses

Associations between air pollutants and mobility were identified from correlations computed between all mobility types reported in the Google COVID-19 Community Mobility Report and concentrations recorded at the selected monitoring sites. Mobility data for the MCMA were obtained from the Google COVID-19 Community Mobility Report (31), which shows changes in visits and length of stay at different places compared to a baseline. The mobility categories reported are retail, grocery, parks, transport stations, residential and workplaces. Further details of how the mobility trends are calculated and descriptions of the place categories can be found on the Google COVID-19 Community Mobility Reports website (31). The subset from 17 March to 21 April 2020 of Google mobility data was selected considering the beginning and end of major mobility changes within the MCMA as shown in Supplementary Figure 1. Significant correlations that passed a hypothesis test with a null hypothesis of 0 correlation are reported with a significance value of α = 0.05, i.e., a high positive significant correlation means that the decrease of an air pollutant is highly correlated with the decrease in mobility.

### Calculation of Potential Health Benefits

The potential health benefits for the MCMA population ascribed to changes in air pollution during the COVID-19 lockdown were addressed using (i) an air quality health index that can reflect the overall improvement in air quality and (ii) calculating changes in the excess risk (ER) of premature mortality. Here, we use the AQHI of Canada to provide insights into the effects of combined changes in NO_2_, O_3_, and PM_2.5_ during the lockdown (32). It measures the air quality on a scale from 1 to 10 which is assigned to a category that describes the level of health risk associated with the index calculated from Low to Very High Health Risk (>10). The AQHI is calculated on a city basis (one or more monitoring sites) and is based on 3-h moving average concentrations as follows (33):

- The average concentrations of O_3_, NO_2_, and PM_2.5_ are calculated at each site for the preceding 3-h with a threshold of 2 out of 3-h at each site, obtaining three pollutant averages for each site. If more than 1 of the preceding 3-h is missing, the site average was not calculated.
- The 3-h city average for each parameter is calculated from the 3-h pollutant averages at the available sites, obtaining 3 city parameter averages. If no sites were available for a parameter, such parameter was not calculated.
- If the three city parameter averages are available, the city AQHI was calculated. Equation (3) shows the AQHI formulation:
  (3) $$\begin{array}{l} AQHI=\;\frac{10}{10.4}(100\;*\;\left(e^{\left(0.000871\;*\;NO_{2}\right)}-1\right)\\ \;+\left(e^{\left(0.000537\;*\;O_{3}\right)}-1\right)+\left(e^{\left(0.000487\;*\;PM_{2.5}\right)}-1\right), \end{array}$$

where all pollutants are entered as 3-h moving average concentrations in ppb for O_3_ and NO_2_ and in μg m^−3^ for PM_2.5_.

Changes in ER-associated with variations in air pollutant concentrations during the lockdown were calculated as follows. Firstly, the relative risk (RR) associated with short-term exposure to criteria air pollutants was calculated using Equation (4) (34):

(4) $$\begin{array}{l} RR_{i}=e^{\left[\beta_{i}\left(c_{i}-c_{i,0}\right)\right]},\;c_{i}>c_{i,0}, \end{array}$$

where *RR*_*i*_ corresponds to the RR of the *i*-pollutant, β_*i*_ is the exposure-response constant indicating the additional health risk per unit of the *i*-pollutant after exceeding a threshold, and *c*_*i*_ is the daily average for pollutant *i*. The World Health Organization (WHO) reported that adverse health effects can occur at any concentration of NO_2_, O_3_, PM, and SO_2_, apart from CO (35). Therefore, we assumed a value of *c*_0_ = 0 for all air pollutants, which implies that pollutant concentrations equal to this value are associated with no excess risk (i.e., RR = 1), while for CO *c*_0_ was equal to 2 mg m^−3^ as reported in (36). The β values used here were 0.038, 0.032, 0.081, 0.13, and 0.048% per μg m^−3^ of PM_2.5_, PM_10_, SO_2_, NO_2_, and O_3_, respectively, while for CO a value of β = 3.7% per mg m^−3^ was used (34). Finally, the ER for the *i*-pollutant was calculated using Equation (5) (37).

(5) $$\begin{array}{l} ER_{i}=RR_{i}-1. \end{array}$$

Additionally, the potential health benefits in the MCMA during the lockdown were quantified by estimated changes in the health burden (Δ*HB*) [number of premature deaths; Equation (6)] due to outdoor air pollution exposure following the methodology reported in (38, 39). The averted health burden (Δ*HB*) during Phase 2 and 3 for the *i*-pollutant was estimated using Equations (6–8).

(6) $$\begin{array}{l} {\Delta HB}_{i}=HB_{i}^{Reference}-HB_{i}^{Lockdown}, \end{array}$$

(7) $$\begin{array}{l} HB_{i}=BM\times ExpPop\times AF_{i}, \end{array}$$

(8) $$\begin{array}{l} AF_{i}=\frac{RR_{i}-1}{RR_{i}}, \end{array}$$

where *BM* is the baseline mortality per 100,000 inhabitants of all age groups, obtained from the Global Burden of Disease study of 2017 (40). *ExpPop* is the exposed population calculated by multiplying the MCMA population by a 75% factor obtained from the Historical Analysis of Population Health Benefits associated with Air Quality in Mexico City during 1990–2015 (41). Finally, *AF* represents the attributable fraction for the RR associated with the *i*-pollutant load during Phases 2–3 and the corresponding reference periods (38).

## Results

### Meteorology in the MCMA

Supplementary Figure 3 shows the monthly anomalies in T, RH and WS within the MCMA for the lockdown Phases 2–3 within the MCMA (March-May 2020). The temperature anomalies suggest a slightly warmer than the average period during March and April between 1 and ~1.5°C, while RH anomalies suggest a decrease only in May. WS anomalies showed similar differences from March to May with the long-term average. Nevertheless, the analysis with the non-parametric Wilcoxon test showed that the observed differences in T, RH and WS were not statistically significant at α = 0.05. Similarly, the WD anomalies did not show significant differences (*p* < 0.05) for the prevailing northerly component between the baseline and lockdown period for March, April and May (dominant WD ~2, ~350, and ~351°, respectively). This suggests that, on average, T, RH, WS and WD were similar during the COVID-19 lockdown in comparison with their long-term averages.

### Modelling of Air Pollutants Time-Series

**Figure 1:**
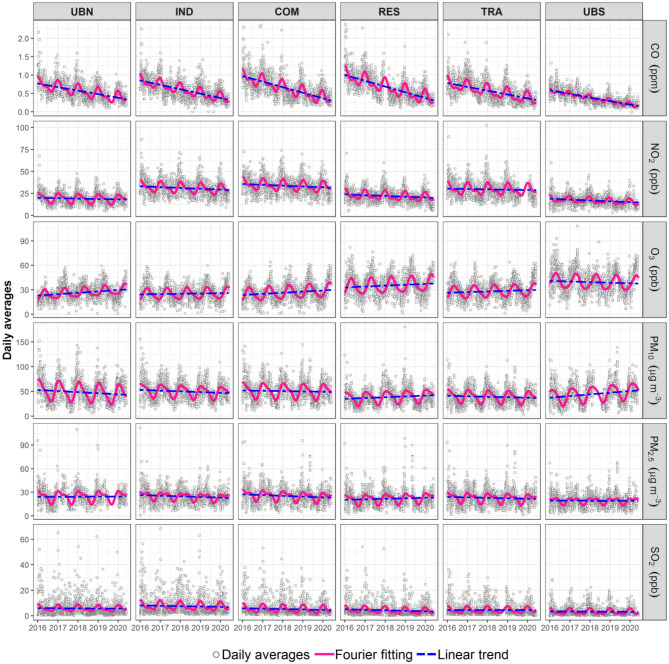
Time-series of criteria air pollutants from 2016 to 2020 at the six selected monitoring sites within the MCMA. The continuous lines show the Fourier fitting and modelled linear trend.

Air pollution within the MCMA peaked during the early 1990s and has decreased ever since as a result of the introduction of emission control policies (42). Although the largest decreases in air pollutant concentrations occurred during the 1990s, some air pollutants still exhibit monotonic trends. Figure 1 shows daily averages for all air pollutants at the representative sites within the MCMA from 2016 to 2020, the adjusted curve using Equation (1) and the linear trend of the time-series. An *F*-test about the model used in this study suggests that the regression computed using truncated Fourier series performs better than the linear model solely (Supplementary Table 3). Furthermore, the residual standard error was <10% for all air pollutant time-series, apart from PM_10_ which showed errors <17% at all sites. This suggests that our model captures the air pollutants behaviour significantly well. It should be noted that the residual standard error is proportional to the magnitudes of observations, and for all air pollutants shows a good fitting.

Overall, all air pollutants exhibited clear seasonal cycles during 2016–2020, while apparent monotonic trends were only observed for CO, NO_2_ and O_3_ (Table 2). All CO decreasing rates were significant (*p* < 0.001) and of similar magnitude (0.1 ppm yr^−1^; 11–15% yr^−1^), while NO_2_ decreased by 1.1–1.2 ppb yr^−1^ (3–6% yr^−1^) at all sites (*p* < 0.05), apart from UBN, where it decreased by 0.3 ppb yr^−1^ (1% yr^−1^; *p* < 0.1). In contrast, O_3_ has increased (*p* < 0.1) between 0.9 and 1.9 ppb yr^−1^ (4–9% yr^−1^), apart from the background sites (UBS and UBN) where no apparent trends were detected likely because of its location on the periphery of the MCMA. PM_10_ increased significantly only at the downwind UBS site (1% yr^−1^), whereas PM_2.5_ decreased between 0.5 μg m^−3^ at TRA and 0.8 μg m^−3^ at COM and IND (~2.5% yr^−1^). The existence of monotonic trends highlight that robust analyses are required to determine whether air pollutants changed within the MCMA during the COVID-19 lockdown.

**Table 2:** Trends for criteria air pollutants expressed in units of concentration during 2016–2020 at the selected monitoring sites within the MCMA.

**Pollutant**	**Annual rate of change**					
	**COM**	**IND**	**RES**	**TRA**	**UBN**	**UBS**
CO (ppm)	−0.1^***^	−0.1^***^	−0.1^***^	−0.1^***^	−0.1^***^	−0.1^***^
NO_2_ (ppb)	−1.1^*^	−1.1^*^	−1.2^*^	−1.1^*^	−0.3	−1.2^***^
O_3_ (ppb)	1.9^***^	0.9	1.6^*^	1.2^***^	1.4	1.5
PM_10_ (μg m^−3^)	0	−0.6	0.4	−0.4	−1.2	3.1^*^
PM_2.5_ (μg m^−3^)	−0.8^*^	−0.8^*^	0.3	−0.5^*^	0.3	−0.2
SO_2_ (ppb)	−0.3	−0.2	−0.3	0	−0.3	−0.1

* *Level of significance p < 0.05*.

*** *Level of significance p < 0.001*.

### The Air Pollutant Anomalies

The analysis of anomalies can provide robust information about odd air pollutants data (43), like those expected during the COVID-19 lockdown. To better isolate the COVID-19 lockdown effects in the MCMA, pollutant hourly anomalies for the Phases 2–3 calculated as described in section Calculation of Anomalies in Air Pollutant Concentrations were compared with those during the 2016–2019 corresponding periods (4-year reference), instead of year-to-year, which can reduce inter-annual variation caused by extraordinary local up to synoptic events (44, 45). Additionally, days with stagnant and heavy rain conditions were excluded from the analysis to minimise rapid weather changes (16, 18, 46). Supplementary Table 4 summarises the minimum, average (mean ± SD) and maximum hourly anomalies during 2016–2019 and Phases 2–3. On average, no significant differences (*p* > 0.05) in the minimum values of anomalies were observed in Phase 2 at all sites for all air pollutants, while the averages were significantly lower, apart from CO and O_3_ that showed increases.

**Figure 2:**
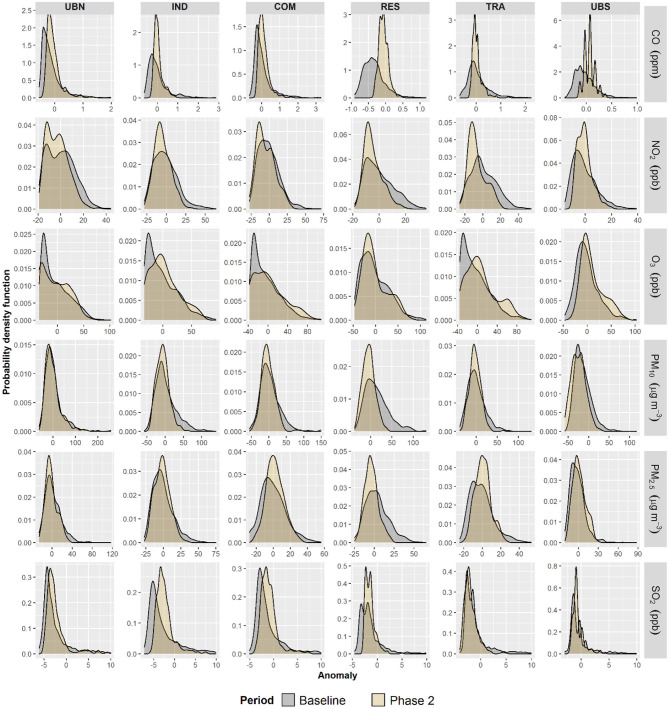
Probability density function plots for hourly anomalies for CO, NO_2_, O_3_, PM_2.5_, PM_10_, and SO_2_ during the COVID-19 lockdown Phase 2 and baseline within the MCMA.

A marked reduction in the maximum anomalies was detected during Phase 2 compared with those during 2016–2019 for all air pollutants, apart from O_3_ and PM_10_. Figure 2 shows the probability density function (PDF) for hourly anomalies calculated during Phase 2. Maximum PDF values for CO, NO_2_, and SO_2_ were higher in Phase 2 than during 2016–2019 due to lower dispersion of anomalies in the upper quartiles. O_3_ anomalies showed a lower maximum PDF at upwind and central sites in Phase 2 than during 2016–2019, while at RES and UBS O_3_ exhibited PDFs consistent with those for CO, NO_2_, and SO_2_. In contrast, PM_10_ and PM_2.5_ PDFs showed less variation at background sites (UBN and UBS) but for central sites, the maximum PDF values occurred during Phase 2.

**Figure 3:**
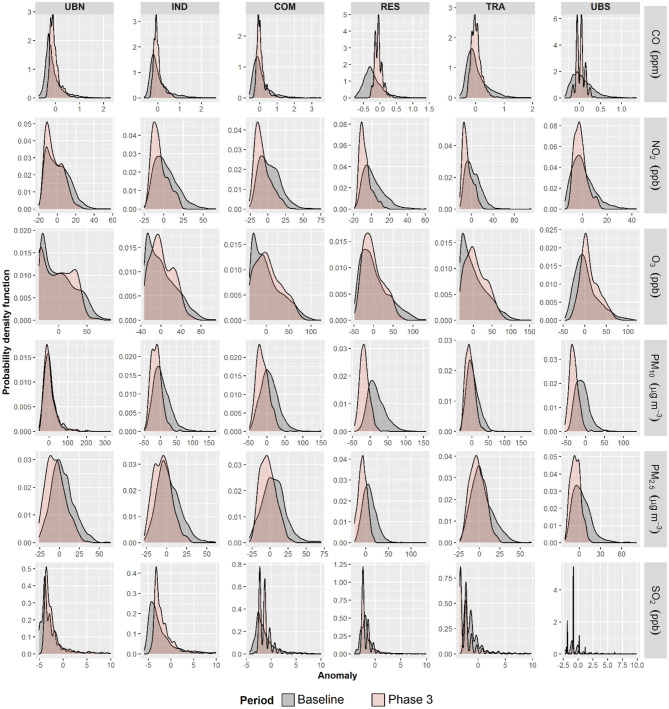
Probability density function plots for hourly anomalies for CO, NO_2_, O_3_, PM_2.5_, PM_10_, and SO_2_ during the COVID-19 lockdown Phase 3 and baseline within the MCMA.

NO_2_, PM_10_, PM_2.5_, and SO_2_ showed higher minimum and lower average anomalies during Phase 3 than in 2016–2019 in good agreement with PDFs values observed in Phase 2, while CO averages were significantly lower in Phase 3 only at UBN and RES (Figure 3). In contrast to Phase 2, O_3_ maximum anomalies decreased in Phase 3 but the average and minimum anomalies were higher than during 2016–2019. PDF showed higher maximums during Phase 3 than from 2016 to 2019 for all pollutants and sites, apart from O_3_. No marked differences in PDFs were observed for all air pollutant anomalies at the UBN in comparison with previous years, suggesting that on average, anomalies were similar in background conditions. However, this was not observed for UBS that showed higher PDFs for negative anomalies in Phase 3 excluding O_3_. Indeed, higher densities of negative anomalies for PM_10_, PM_2.5_, NO_2_, and SO_2_ were observed in Phase 3 at most of the sites. This suggests that clear reductions in emissions of these air pollutants occurred within the MCMA only in Phase 3 under the total restriction of all non-essential activities. Furthermore, the differences in PDF distributions between Phase 2 and 3 suggest marked spatial variation in air pollutants concentrations within the different environments of MCMA.

### Air Pollutants Diurnal Cycles

The air pollutant diurnal cycles arise from changes in meteorology and pollutant emissions, mostly from fossil fuel combustion (47–49). The lockdown measures within the MCMA included restrictions to population mobility, industrial production and office and leisure activities which may have reduced air pollutant emissions. Here, the effect of restrictions on normal anthropogenic activities in the MCMA context is analysed by comparing de-trended air pollutant diurnal cycles constructed from hourly anomalies during Phases 2–3 with the corresponding anomalies during 2016–2019. Changes in the amplitude values (AVs) of diurnal cycles during the lockdown may reflect local changes in emission sources (50). AVs of diurnal cycles for all criteria air pollutants were calculated as the difference trough-to-peak and the significance of changes in AVs was evaluated through Welch's *t*-tests.

**Figure 4:**
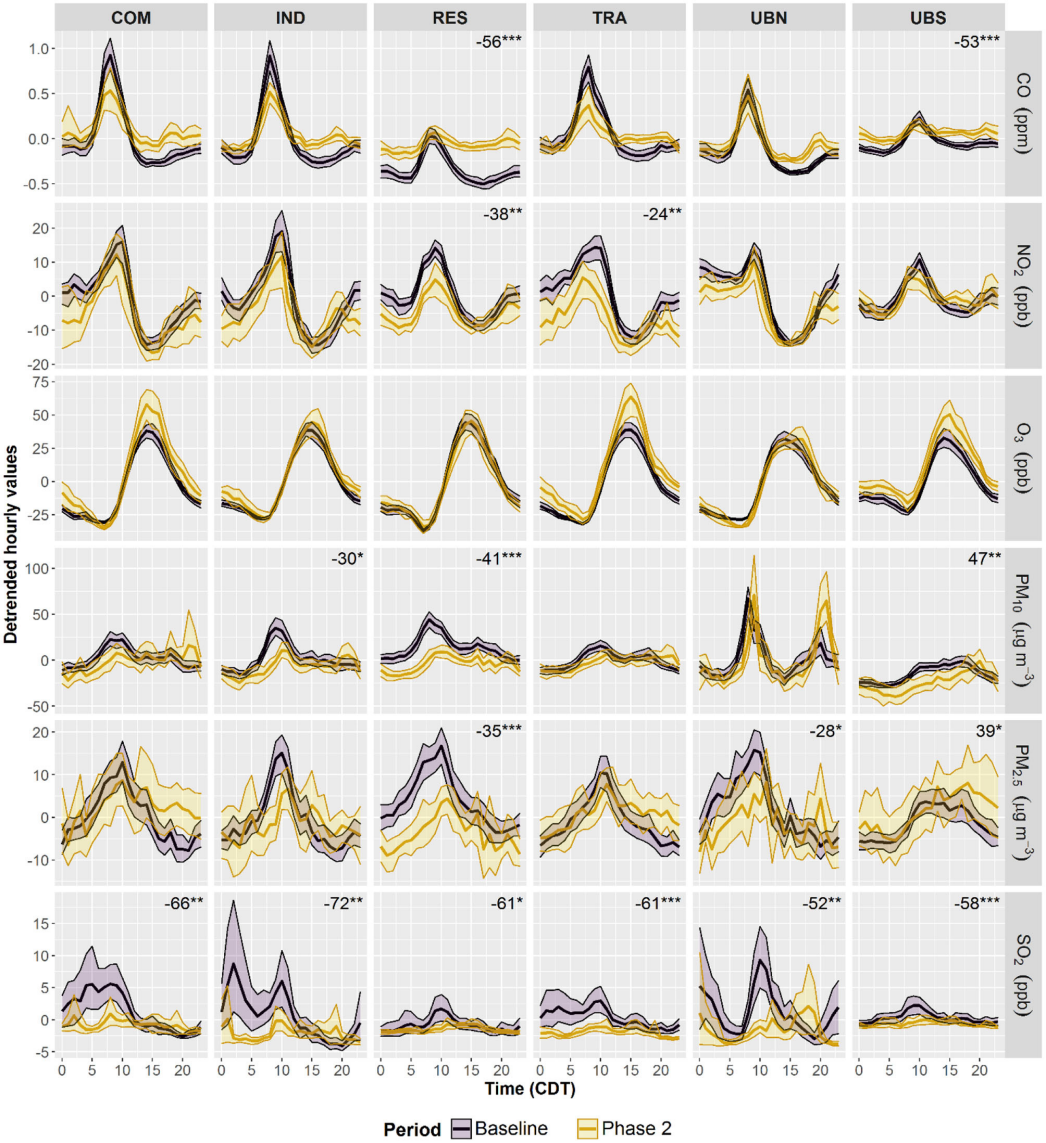
Average diurnal cycles within the MCMA during 2016–2019 and lockdown Phase 2. De-trended diurnal cycles were constructed by subtracting daily averages modelled with Equation (1) from hourly averages to remove the impact of long-term trends. The shading shows the 95% confidence intervals of the average, calculated through bootstrap re-sampling for 1,000 iterations. The numbers show significant changes in diurnal amplitudes, through-to peak, expressed as percentage with 2016–2019 cycles as baseline. *Level of significance *p* < 0.05. **Level of significance *p* < 0.01. ***Level of significance *p* < 0.001.

**Figure 5:**
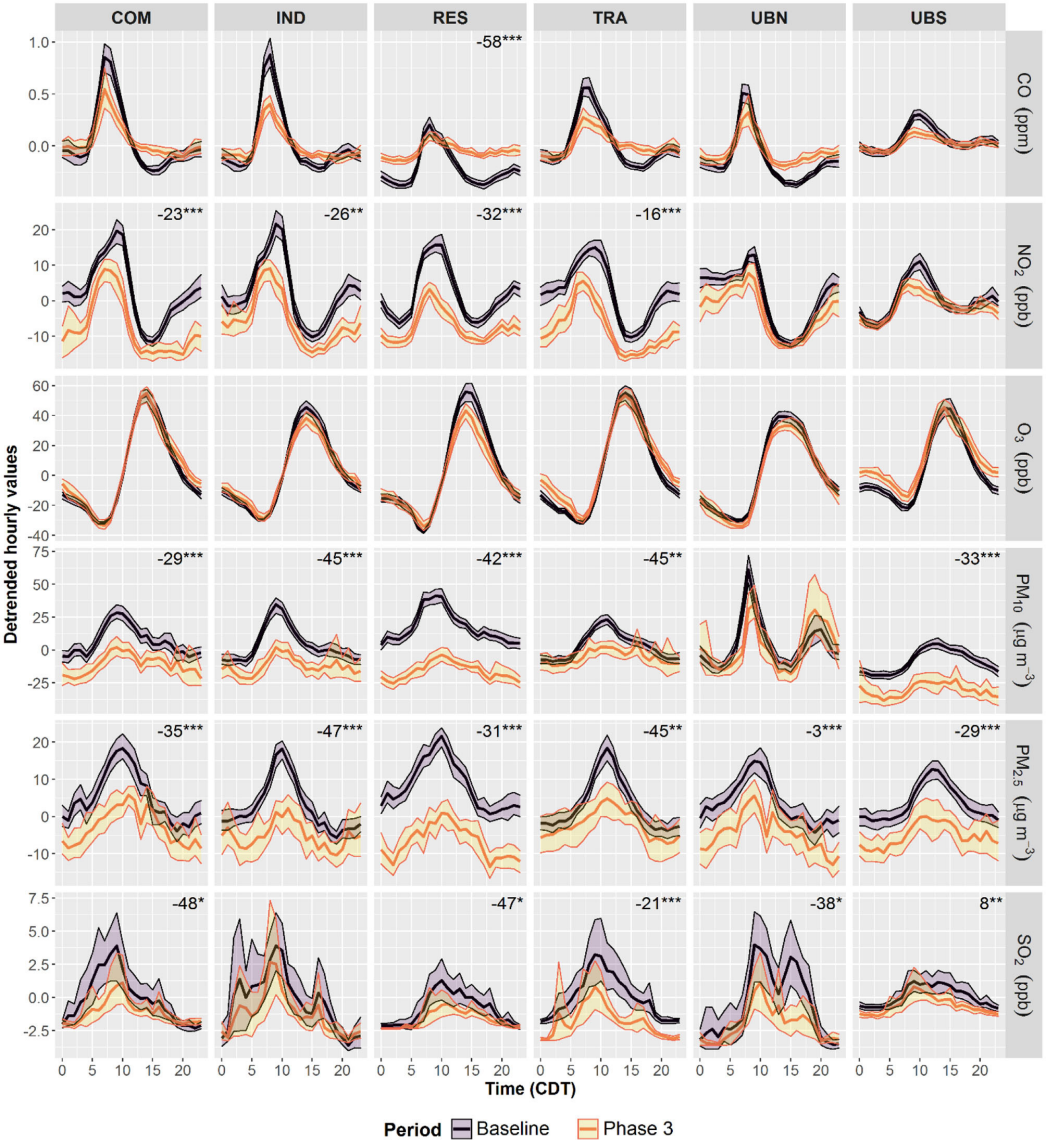
Average diurnal cycles within the MCMA during 2016–2019 and lockdown Phase 3. De-trended diurnal cycles were constructed by subtracting daily averages modelled with Equation (1) from hourly averages to remove the impact of long-term trends. The shading shows the 95% confidence intervals of the average, calculated through bootstrap re-sampling for 1,000 iterations. The numbers show significant changes in diurnal amplitudes, through-to peak, expressed as percentage with 2016–2019 cycles as baseline. *Level of significance *p* < 0.05. **Level of significance *p* < 0.01. ***Level of significance *p* < 0.001.

Figure 4 shows that during Phase 2, changes in AVs were evident mostly at sites in the MCMA centre, while background sites on the MCMA periphery showed less marked changes. SO_2_ AVs exhibited significant (*p* < 0.05) decreases at all sites between 58 and 72% probably due to the low concentrations typical within the MCMA. O_3_ AVs did not change at any site in Phase 2 despite decreases in NO_2_ of 38 and 24% at RES and TRA, respectively. RES was the only site that showed significant decreases in AV for all air pollutants, apart from O_3_ which did not change, and only UBS showed increases both in PM_10_ and PM_2.5_ AVs. During Phase 3, most air pollutants AVs showed decreases within the MCMA (Figure 5), apart from O_3_ which did not change. As in Phase 2, only RES showed significant (*p* < 0.05) decreases in AVs for almost all air pollutants and it was the only site where CO decreased (58%) in Phase 3. NO_2_, PM_10_, and PM_2.5_ AVs decreased at all sites in the MCMA centre between 16 and 32, 29–45, and 31–47%, respectively. SO_2_ AVs also decreased in Phase 3, apart from IND, between 21 and 48%, but increased at UBS (8%). The observed changes in AVs suggest that reductions in air pollutant emissions were generalised and significant within the MCMA only after the restriction of all non-essential activities.

### Net Changes in Air Pollutants During the Lockdown

**Figure 6:**
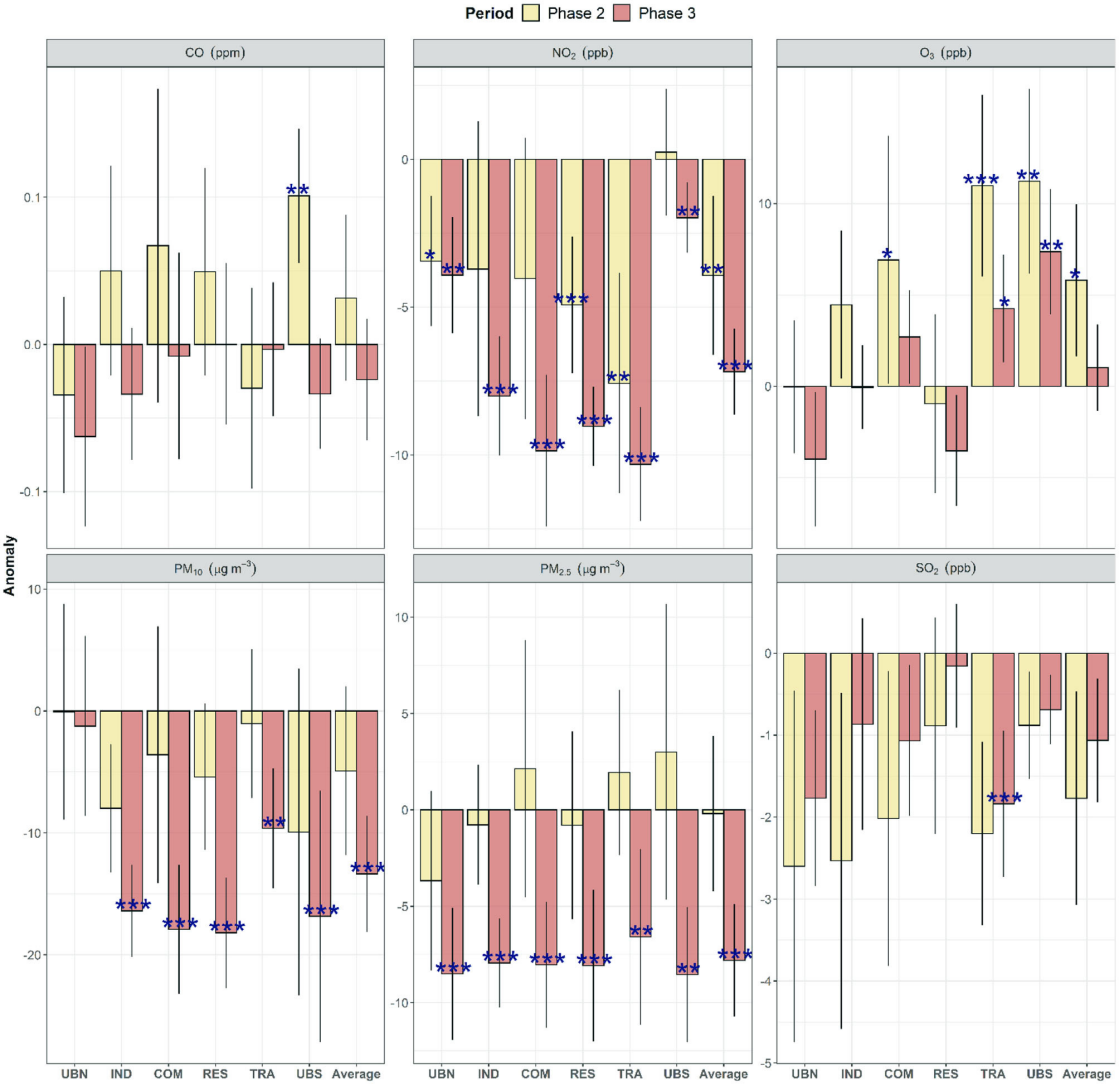
Net changes in anomalies of criteria air pollutants during lockdown Phases 2–3 in comparison to 2016–2019 anomalies as baseline within the MCMA. The stars show significant changes during the COVID-19 lockdown calculated with the non-parametric Wilcoxon test, while the vertical bars show the 95% confidence intervals. *Level of significance *p* < 0.05. **Level of significance *p* < 0.01. ***Level of significance *p* < 0.001.

The overall changes in air pollutant concentrations during the COVID-19 lockdown within the MCMA were determined by comparing hourly anomalies calculated for the lockdown period with those from the 2016–2019 corresponding period. The significance of the obtained changes was calculated with the non-parametric Wilcoxon test. Figure 6 shows that during Phase 2, only NO_2_ decreased significantly (*p* < 0.05) between 3 ppb (10%) and 8 ppb (23%) at UBN and TRA, respectively, with an average decrease of 4 ppb (around 20%). By contrast, significant increases (*p* < 0.05) were observed for O_3_ ranging from 7 ppb (16%) at COM to 11 ppb (40%) at TRA and UBS, likely because of reduced O_3_ titration by NO_2_. CO only increased at UBS (0.1 ppm, 4%), whereas SO_2_, PM_10_, and PM_2.5_ did not exhibit significant (*p* > 0.05) changes.

During Phase 3, NO_2_ decreased (*p* < 0.05) significantly between 7 and 10 ppb (average decrease of 43%) at most sites, which are larger decreases than in Phase 2. Similarly, significant decreases (*p* < 0.05) were observed at all sites both for PM_10_ and PM_2.5_ during Phase 3, apart from UBN. Nevertheless, while PM_2.5_ decreased consistently by 7–8 μg m^−3^ (20%), large spatial variability was observed for PM_10_ which decreased between 10 and 18 μg m^−3^, with an average decrease of 32%. SO_2_ only decreased significantly at TRA (2 ppb, 31%) likely ascribed to reduced emissions from heavy-duty vehicles and buses. O_3_ showed large spatial heterogeneity in Phase 3, with significant increases (*p* < 0.05) seen only at TRA (4 ppb, 14%) and UBS (7 ppb, 19%). Our results allow us to hypothesise that the decreases observed in concentrations of primary pollutants during Phase 3 within the MCMA can be ascribed to the strict lockdown focused on controlling the COVID-19 spread.

To better observe the effects of long-term trends and rapid weather events on air pollution, we compared changes in air pollutants calculated from raw observations with those from anomalies including and excluding stagnant conditions and heavy rain events during Phase 2 (Supplementary Figure 4) and Phase 3 (Supplementary Figure 5). The largest differences were observed clearly for CO, with overestimated changes > 10 times in both Phase 2 and 3, due to the influence of long-term trends. For the other air pollutants, most of the changes during Phase 2 could have been overestimated between 0.5 and 2 times without accounting for the rapid weather changes. Similarly, an overestimation generalised of around 2 times was observed for all air pollutants, excluding CO, during Phase 3. In particular, the overestimation of O_3_ increases may mislead the design of future control policies by making it difficult to scale emission inventories and affecting photochemical models performance.

### Changes in the NO_2_ and CO Columns Over the MCMA

**Figure 7:**
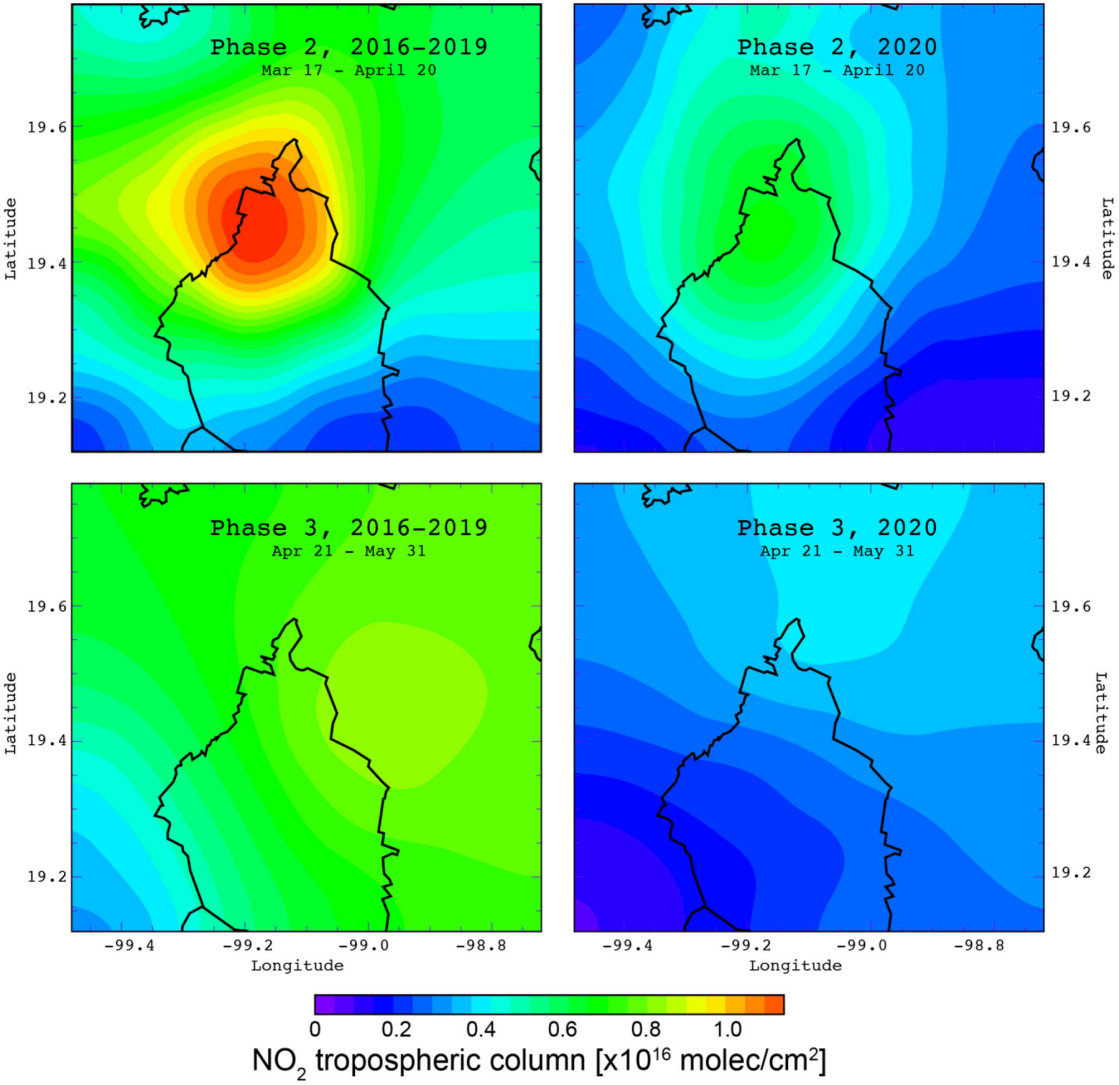
NO_2_ tropospheric column distribution maps constructed using satellite-based data from OMI over the MCMA during 2016–2019 (baseline) and lockdown Phases 2–3.

Figure 7 shows averaged NO_2_ tropospheric column distribution maps constructed using satellite-based data above the MCMA for the Phases 2–3 and corresponding periods during 2016–2019. Reductions in the amount of NO_2_ molecules present in the atmospheric column were observed for both lockdown Phases, with the largest decreases observed in Phase 3. Spatially, the largest decrease was seen in the north of MCMA where the maximum NO_2_ column values decreased from ~1.1 × 10^16^ to ~0.8 × 10^16^ molecules (molec.) cm^−2^ from 2016–2019 to Phase 2 (−30%), and from ~0.8 × 10^16^ to ~0.4 × 10^15^ molec. cm^−2^ during 2016–2019 to Phase 3 (−50%). The MCMA central and southern regions exhibited decreases of between 10 and 40% in the NO_2_ column during Phase 3, which aligns well with the decrease calculated from NO_2_ surface observations. The difference in NO_2_ decreases between Phase 2 and 3 can be explained by a gradual reduction in motor vehicle circulation during Phase 2, while large reductions in NO_X_ emissions from motor vehicles and other combustion sources occurred in Phase 3 because of the total lockdown.

**Figure 8:**
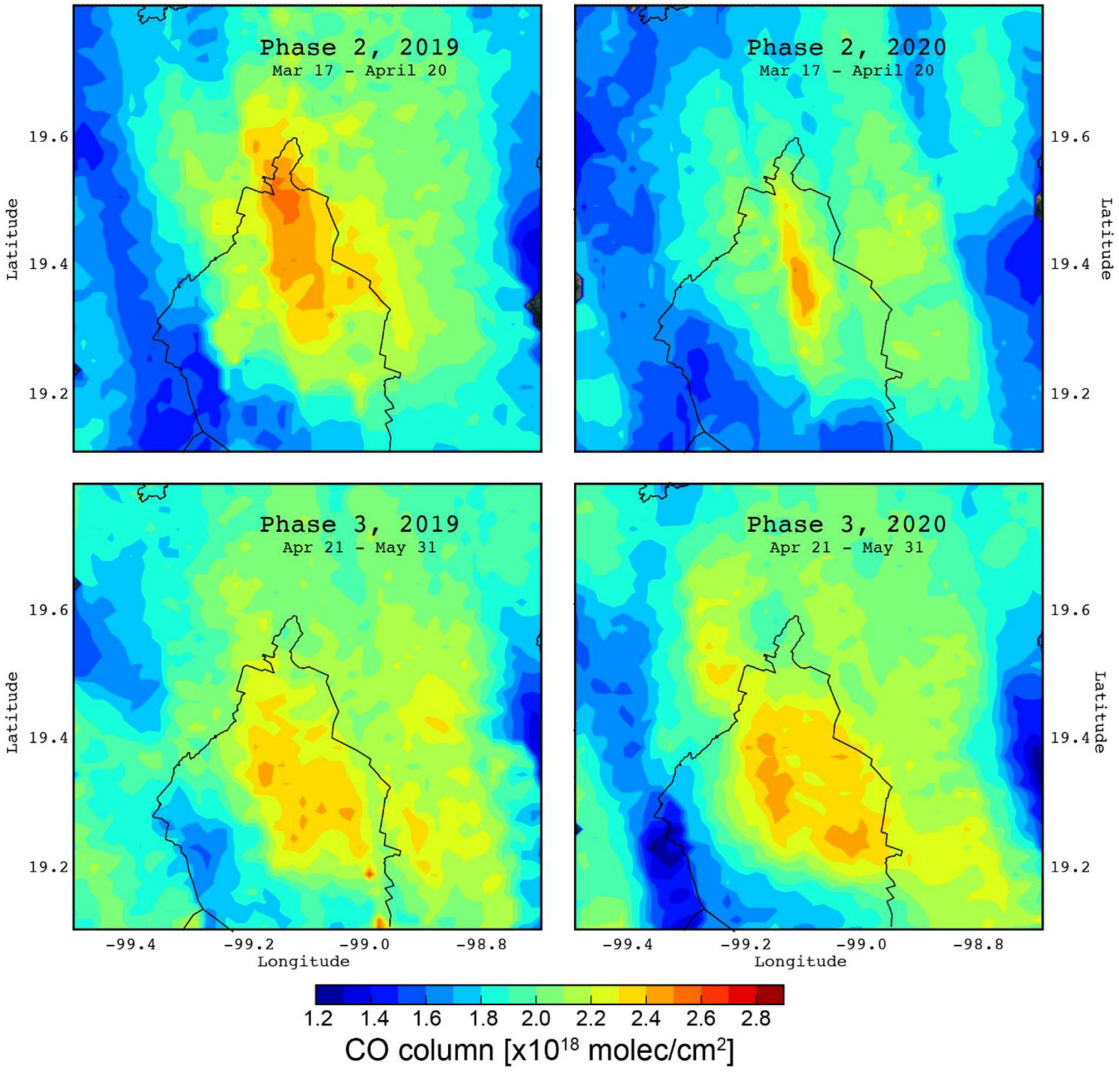
Averaged CO column distribution maps constructed from satellite-based data from TROPOMI over the MCMA during lockdown Phases 2–3 of 2020 (right) with respect to the previous year (left).

Figure 8 shows averaged CO column distribution maps from satellite-based data over the MCMA during the lockdown Phases 2–3 and the corresponding periods in 2019. Differences between the CO columns of 2019 and Phase 2 were noticed for the central and northern MCMA regions, which corresponded to decreases of around 20 and 33%, respectively. Such differences were less evident for the southern MCMA region. No marked changes in the CO column were seen during Phase 3 compared to 2019 over most of the MCMA. Only over Phase 3, the satellite-based data are consistent with surface observations that suggest no significant changes in CO. Stremme et al. (51) reported that the CO column over MCMA is occasionally influenced by biomass burning events, which could have biased the satellite data during Phase 2 because only data for 2019 was available to use as a baseline. Although the CO is emitted mostly by motor vehicles within the MCMA (48), it is not clear why no reductions were observed during the lockdown despite reduced road traffic. A plausible explanation could be an increase in CO emissions during the lockdown from enhanced domestic liquid petroleum and natural gas burning because of the stay-at-home order, however, further investigation is required to clarify it.

### Changes in AQHI and Excess Risk

The AQHI was used here because it can provide information about the additive effects of O_3_, NO_2_, and PM_2.5_ which are known to harm human health. Supplementary Figure 6 shows the boxplot of maximum daily AQHI values during 2016–2020 and Table 3 lists the calculated changes. During Phase 2, all sites showed similar maximum AQHI daily values of 15–16. Nevertheless, only the decreases (ΔAQHI = −3) at RES, IND and COM were significant (*p* < 0.05). The maximum AQHI daily values also decreased significantly (*p* < 0.05) at all sites during Phase 3 from 2 to 6 units (average = 4). The decrease in the maximum daily AQHI values during the lockdown suggest that, despite increases in O_3_, the reductions in NO_2_, and PM_2.5_ could represent a potential benefit for the MCMA air quality.

**Table 3:** Averages and changes in maximum daily AQHI values during 2016–2019 and lockdown Phases 2–3 by monitoring site within the MCMA.

**Period**	**Site**	**2016–2019**	**Lockdown**	**ΔAQHI^a^**
Phase 2	UBS	15	16	1
	UBN	17	16	−1
	TRA	17	16	−1
	RES	18	15	−3^***^
	IND	19	16	−3^**^
	COM	19	16	−3^*^
	Average	17	15	−2^*^
Phase 3	UBS	18	12	−6^***^
	UBN	18	16	−2^***^
	TRA	20	15	−5^***^
	RES	21	15	−6^***^
	IND	22	16	−6^***^
	COM	22	17	−5^***^
	Average	19	15	−4^***^

a *Levels of significance determined with the non-parametric Wilcoxon test*.

* *Level of significance p < 0.05*.

** *Level of significance p < 0.01*.

*** *Level of significance p < 0.001*.

We calculated the ER of premature mortality for each criteria air pollutant to evaluate possible health benefits within the MCMA during the lockdown as a result of changes in pollutant concentrations (Table 4; Supplementary Figure 7). A significant decrease (*p* < 0.01) in the ER of premature mortality was observed during Phase 2 only for NO_2_ (27%) while for O_3_ it increased by 25%. During Phase 3, the ER of premature mortality showed significant decreases (*p* < 0.01) for NO_2_, PM_10_, PM_2.5_, and SO_2_, with the largest decrease observed for NO_2_ (36%) and the lowest for PM_10_ (24%). Finally, the health assessment conducted suggested that around 588 deaths were averted during the lockdown, explained by decreases in the health burden from the reference period to the lockdown. This decrease in health burden can be ascribed to reduced population exposure to outdoor air pollution during the lockdown. Nevertheless, while around 152 deaths were averted during Phase 2, an estimate of averted deaths of 436 was found for Phase 3. This is in line with a decline in the ER of premature death larger during Phase 3 than for Phase 2. Together the AQHI and ER of premature mortality analyses can confirm that a significant decrease in potential outdoor exposure to air pollutants occurred in the MCMA as a result of the lockdown measures and health benefits that occurred because of these measures.

**Table 4:** Average excess risk within the MCMA for each criteria air pollutant during 2016–2019 (Reference) and COVID-19 lockdown Phases 2–3.

**Phase**	**Pollutant^a^**	**Period**		**Change in %**	**Significance^b^**
		**Reference**	**Lockdown**		
Phase 2	NO_2_	0.0632	0.0463	−27	^***^
	O_3_	0.0335	0.0418	25	^**^
	PM_10_	0.0167	0.0151	−10	
	PM_2.5_	0.0096	0.0094	−3	
	SO_2_	0.0109	0.0057	−48	
Phase 3	NO_2_	0.0660	0.0422	−36	^***^
	O_3_	0.0399	0.0427	7	
	PM_10_	0.0164	0.0125	−24	^***^
	PM_2.5_	0.0111	0.0081	−27	^***^
	SO_2_	0.0082	0.0048	−42	^**^

a *No average excess risk for CO was calculated because its concentrations did not exceed the limit of 2 mg m^−3^ considered as safe (36)*.

b *Levels of significance determined with the non-parametric Wilcoxon test*.

** *Level of significance p < 0.01*.

*** *Level of significance p < 0.001*.

## Discussion

### The Origin of Changes in Air Pollutants During the Lockdown

Urban mobility showed significant reductions worldwide during the lockdowns (Supplementary Table 1). Within the MCMA, mobility in all categories decreased between 50 and 65% during Phase 2 (Supplementary Figure 1), apart from the residential which increased by 25%, and all stabilised in Phase 3. This suggests that during Phase 3, the population spent longer periods in residential environments because of the restrictions on mobility and labour activities (31). A generalised correlation was observed between mobility and the decreases for NO_2_ (*p* < 0.05), contrasting with O_3_ which showed negative correlations (Supplementary Table 5). This may confirm that the reductions observed in NO_2_ of up to 60% within the MCMA during Phase 3 were ascribed to the overall reduction of motor vehicles circulation (21, 52).

Lower emissions from fossil fuel combustion by motor vehicles can be confirmed by decreases in average petrol and diesel sales within the MCMA during the lockdown phases compared to 2016–2019 sales (Supplementary Figure 8) (53). Furthermore, the largest decrease in petrol consumption was observed during Phase 3, thus suggesting a decrease in VOCs emissions but only from incomplete combustion. Although CO also showed correlations with mobility at some sites, the changes in CO were non-significant, despite a reported 28% reduction in CO emissions (52). This could be due to increased domestic burning of natural and liquified petroleum gas because of the time spent at home, as suggested by the mobility analysis, however, further analyses are required to clarify this. SO_2_, PM_10_ and PM_2.5_ showed less marked correlations despite the reduction in diesel consumption as motor vehicles are not their major source of emissions.

O_3_ formation in the MCMA central-southern regions is VOC-limited but NO_X_-limited on the outskirts (54, 55). The reductions in NO_X_ emissions from motor vehicles could thus explain the observed increases in O_3_ during Phase 2; as a result of reduced O_3_ titration (*NO*+ *O*_3_ = *NO*_2_ + *O*_2_). VOC emissions did not present significant reductions since some activities related to area emissions such as residential sources did not decrease or even increase during Phase 2. Marked decreases in petrol and diesel sales within the MCMA during the lockdown suggest a decline in VOC emissions from incomplete combustion accentuated in Phase 3 (Supplementary Figure 8). The generalised stabilisation of O_3_ as shown by the averaged anomalies during Phase 3, suggests that combined reductions of NO_X_ and VOC emissions occurred when vehicle emissions troughed and most of the stationary and area emission sources other than domestic were out of operation. This hypothesis can be supported by studies addressing the O_3_ weekend effect (56, 57) and the power plant influence located north of the MCMA (58), which have reported that in VOC-limited conditions, reductions in NO_X_ emissions even combined with reductions in VOCs, would lead to increments in the O_3_ levels.

The MCMA exhibits currently low SO_2_ levels because since 2009 the diesel sold in this is ultra-low sulphur (42, 48), which may explain that SO_2_ showed the lower changes among all air pollutants during the lockdown. However, the decrease in SO_2_ seen at TRA may suggest that additional reductions in SO_2_ emissions from heavy-duty vehicles, which currently contribute with around 21% of total emissions, could be obtained by increasing the availability of ultra-low sulphur diesel in other areas of central Mexico. Indeed, it is required for the operation of less polluting heavy-duty technologies such as EPA−10 and Euro VI. On the other hand, reductions in the operation of stationary and area sources appeared to have no significant effect on SO_2_ levels despite contributing to 33 and 46% of total SO_2_ emissions (48). PM_10_ and PM_2.5_ only decreased significantly in Phase 3 when their major emitters, stationary and area sources, were out of operation thus suggesting that to reduce their levels air quality policies must include undoubtedly these sources.

### Air Pollutants Changes in the MCMA in the International Context

Marked decreases in NO_2_ emissions of up to 30% were observed worldwide due to reductions in transport activities implemented to control the COVID-19 pandemic in urban centres (59). In the MCMA, NO_2_ concentrations decreased between 10 and 50% during the lockdown phases in good agreement with reduced energy and fuel demand as observed in other cities, confirming that motor vehicles represent the major share of NO_2_ urban emissions (48). Increases in O_3_ observed during the lockdown within the MCMA are consistent with those in major urban areas around the world where O_3_ production has been determined as VOC-limited. For instance, this behaviour was observed in Barcelona, Spain (10), São Paulo, Brazil (60), and Delhi, India (61), and overall was ascribed to large NO_X_ emission reductions in VOC-limited environments. The O_3_ observations during the COVID-19 pandemic within the MCMA suggest that measures focused only on reducing NO_X_ or VOCs–NO_X_ combined emissions can lead to increases in O_3_. Nevertheless, qualitative and quantitative data of ambient VOC levels are required to gain a thorough understanding of the effect of precursor emission reductions on O_3_ levels.

Area sources represent the major share of PM_10_ (58%) and PM_2.5_ (47%) total emissions within the MCMA, followed by motor vehicle sources with 29 and 36%, respectively. These contributions aggregated, account for 87 and 83% of PM_10_ and PM_2.5_ total emissions, which highlights that both pollutants decreased at all sites only after the combined total restriction of non-essential commercial, industrial and mobility activities occurred during Phase 3 (62). This is consistent with the change in PM_10_ levels observed in Rio de Janeiro by Dantas et al. (63) who reported that PM_10_ decreased only when most of the lockdown measures were in force. That said, Wang et al. (64) reported that pollution events occurred in 9 Chinese cities due to unfavourable meteorology, despite decreases in PM_2.5_ emissions during the lockdown. Thus, the observed reductions both in PM_10_ and PM_2.5_ within the MCMA suggest that only combined strategies targeting different emission sources can be effective for reducing PM. Only TRA showed a decrease in Phase 3 in SO_2_, highlighting the fact that MCMA is a low SO_2_ city with the major contribution of heavy-duty vehicles. An opposite scenario was experienced in northern India where upwind emissions from power plants increased the SO_2_ levels due to the enhanced domestic energy demand because of the lockdown (45).

Several studies have addressed changes in population mortality due to air pollution exposure during the lockdown. Here, we used city average relative risks (RR) for each pollutant by lockdown phase with the aim to include an estimation of averted deaths relative to the lockdown measures applicable. In line with the estimated reductions in health burden in five Indian cities reported by Kumar et al. (39), we can remark that air pollution-related deaths declined during the lockdown period. We estimated a higher number of averted deaths at city scale than that of Kumar et al. (39) likely because they only studied the impact of PM_2.5_. Similarly, Sharma et al. (45) reported that the average decrease in the ER premature mortality for all air pollutants in India observed under lockdown could avert around 0.65 million deaths in a year if pollutant concentrations decrease. For the MCMA, around 3,385 deaths per year could be averted if the ER of premature mortality observed during Phase 3 was maintained, while this estimated would decrease to 1,852 averted deaths with the ER of premature mortality decrease observed during Phase 2. In conclusion, these estimates suggest that future air quality policies can be oriented using the information gathered during the COVID-19 lockdown to protect the health of the MCMA inhabitants.

### Contribution and Limitations

The dry-hot season between March and May in the MCMA is characterised by recurrent pollution episodes of O_3_, PM_10_, and PM_2.5_ (49), associated with calm winds, low RH and low-temperature fluctuations at synoptic scale (16). For instance, during 10–17 May 2019, pollutant emissions from wildfires in central and southern Mexico affected severely the air quality within the MCMA during the occurrence of a high-pressure system (65). In an effort to reduce the O_3_, PM_10_, and PM_2.5_ levels, the MCMA local authorities implemented extraordinary measures by the beginning of the episode such as reducing motor vehicle circulation, restricting the maintenance and operation of LP gas plants, suspension of highly polluting industries and all cleaning and maintenance activities. Despite such measures, the episode lasted for 5 more days after their implementation. This event highlighted how extreme weather conditions in the MCMA may obscure the benefits of extraordinary measures introduced to reduce air pollutant emissions and should be taken into account when conducting public policies evaluation.

Indeed, air pollution episode action-plans may yield significant benefits in terms of primary and secondary pollutants load in the airshed during these episodes. However, the potential benefits of those plans may not be properly assessed during pollution episodes because, in most cases, these were triggered by external drivers such as strong high-pressure systems or forest fires. The air pollutant emission reductions during the lockdown Phase 3 in the MCMA were larger than any achievable reduction resulting from the implementation of extraordinary measures, such as those implemented during the 2019 pollution episode (65). Thus, the COVID-19 lockdown allowed us to assess the potential benefits of potential public policies on air quality under non-episodic conditions.

We also aimed to minimise the influence of long-term trends and inter-annual variability on air pollutant concentrations during the COVID-19 lockdown since some pollutants exhibited monotonic trends. The comparison between changes from raw data with those from data including and excluding stagnant and heavy rain events during Phases 2–3 is shown in Supplementary Figures 4, 5, respectively. CO exhibited the largest differences between estimated changes with an overestimation of changes >10 times for raw data during both phases. The other air pollutants showed less variability in Phase 2, with most overestimations ranging between 0.5 and 2 times for raw data. During Phase 3, a generalised overestimation of around 2-fold was observed for all air pollutant changes, apart from CO. On the other hand, a comparison between changes in air pollutants calculated using the 4-year baseline with those year-to-year showed differences <10% for all pollutant and sites, apart from O_3_, PM_10_, and PM_2.5_ at UBN (Supplementary Table 6). This is explained by large inter-annual pollutant variability at UBN. Therefore, the 4-year baseline used here may both minimise the impact of inter-annual variability and long-term trends (42, 44).

Since the photochemistry is a non-linear process, it requires a set of model sensitivity analyses and field experiments to quantify the impacts on emission reductions on air pollutant concentrations. In particular, the overestimation of O_3_ increases may mislead the design of future control policies by making it difficult to scale emission inventories and affecting the performance of photochemical models. In this sense, the COVID-19 lockdown measures reduced anthropogenic activities providing an opportunity to observe a real-life experiment for emission reductions and their effects on the air quality in the MCMA. By precisely quantifying the reductions and air quality changes during the lockdowns, it is possible to calibrate photochemical models and scale the emissions inventory to evaluate other policy strategies for improving the air quality in the studied area. We suggest that those strategies can be applied in other regions around the world with similar air quality problems.

Finally, it is important to bear in mind that our findings can be limited to the analysis of data available in the urban environments selected. While the monitoring sites selected in the MCMA may represent typical environments existing in most cities across the world, future analyses must include a larger number of monitoring sites to better capture the air pollution spatial and temporal variation. Also, the public health benefits related to reduced air pollution cannot be extrapolated to other cities and can be subject to uncertainty due to the variables used in the calculation process. Nevertheless, such analyses may contribute to a better understanding of the impact of extraordinary public policies on air pollution and public health.

## Conclusions

The MCMA megacity has a long history of air pollution problems. MCMA local authorities have regularly implemented and updated measures to reduce pollutant emissions since the early 1990s, however, the COVID-19 lockdown represents a unique opportunity to evaluate a what-if scenario. While most of the published studies about air pollution changes during the lockdown periods have based their results on raw observations, we presented results based on air pollutant anomalies which are more resistant to meteorological influences and long-term trends influences. Hypothesis tests at a confidence level of α = 0.05 confirmed that our models fitted more accurately the air pollutant data than means. Therefore, the calculated differences in air pollutant anomalies provided confident evidence of the major drivers of air pollutant changes during the lockdown. Monitoring sites representative of different environments were selected based on air pollutant data captured during the lockdown within the MCMA. Although the selected sites showed consistent results for all air pollutants and with those derived from remote sensing data, additional data for the CO satellite column could help to confirm the non-significant changes.

Our results suggest that while NO_2_ decreased significantly because of reduced motor vehicle emissions, other criteria pollutants require more stringent emission control policies for their abatement within the MCMA. These results confirm that to reduce O_3_ levels within the MCMA, future strategies must be introduced to reduce VOC emissions from other sources rather than just from motor vehicles. Overall, air quality improved during the lockdown in response to reduced NO_2_ and PM_2.5_ emissions despite the increases in O_3_ levels. A health assessment conducted suggested an estimate of around 588 averted deaths as a result of reduced air pollution during the lockdown. The analysis presented here could be extended to a regional scale to assess the influence of air pollutant emission sources on the MCMA outskirts and surrounding states. Finally, the measures implemented during the COVID-19 lockdown provide valuable information to reduce air pollution through a range of abatement strategies for emission other than from motor vehicles, which have received the most attention, particularly in economically developing countries.

## Data Availability Statement

Publicly available datasets were analyzed in this study. This data can be found at: http://www.aire.cdmx.gob.mx/default.php.

## Author Contributions

IH-P and MG conceived the study, participated in its design, and coordination. IH-P, SV, VA, CR-C, WS, LR-S, AG-R, and MG collected the data, entered study data, assisted in data analysis, and interpretation of study results. IH-P, SV, VA, CR-C, WS, MG, and LR-S performed final analyses and co-drafted the manuscript. All authors read, revised, and approved the final manuscript.

## Conflict of Interest

The authors declare that the research was conducted in the absence of any commercial or financial relationships that could be construed as a potential conflict of interest.

## Abbreviations

AF: Attributable fraction
AQHI: Air quality health index
ASI: Air Stagnation Index
AV: Amplitude value of diurnal cycle
CO: Carbon monoxide
COM: Commercial site
COVID-19: Coronavirus disease
ER: Excess risk
EPA: US Environmental Protection Agency
GPM: Global Precipitation Measurement
HB: Health burden
IND: Industrial site
MCMA: Mexico City Metropolitan Area
Molec: molecules
NO_2_: Nitrogen dioxide
NO_X_: Nitrogen oxides
O_3_: Ozone
OMI: Ozone Monitoring Instrument
PDF: Probability density function
PM_2._5: Particulate matter with aerodynamic diameter <2.5 μm
PM_10_: Particulate matter with aerodynamic diameter <10 μm
RES: Residential site
RH: Relative humidity
RR: Relative risk
SD: Standard deviation
SIMAT: Atmospheric Monitoring System of the Mexico City government
SO_2_: Sulphur dioxide
T: Temperature
TRA: Traffic site
TROPOMI: Tropospheric Monitoring Instrument
UBN: Northern background site
UBS: Southern background site
VOC: Volatile organic compounds
WHO: World Health Organization
WD: Wind direction
WS: Wind speed.
