# Supplementary material for: Impact of the COVID-19 Lockdown on Air Quality and Resulting Public Health Benefits in the Mexico City Metropolitan Area
Source: Front Public Health. 2021 Mar 25;9:642630. doi: 10.3389/fpubh.2021.642630 (PMC8026884; doi:10.3389/fpubh.2021.642630)
Supplement: Supplementary file 1 [file Table_1.DOCX]

Supplementary Material

Impact of the COVID-19 lockdown on air quality and resulting public health benefits in the Mexico City Metropolitan Area

**Iván Y. Hernández Paniagua^1,^*, Sergio Ivvan Valdez^2^, Victor Almanza, Claudia Rivera Cárdenas^1^, Michel Grutter^1^, Wolfgang Stremme^1^, Agustín García Reynoso^1^, Luis Gerardo Ruiz Suárez^3^**

^1^Centro de Ciencias de la Atmósfera, Universidad Nacional Autónoma de México, Ciudad de México, México.

^2^CONACYT-Centro de Investigación en Ciencias de Información Geoespacial, Ciudad de México, México.

^3^Instituto Nacional de Ecología y Cambio Climático, Ciudad de México, México.

*** Correspondence:**Corresponding Author
ivan.hernandez@atmosfera.unam.mx

## Supplementary Tables

**Table S1.** Average changes in urban mobility by category within some of the greatest metropolitan areas around the world during the respective COVID-19 lockdowns.

| Region | % of change in mobility to place categories | | | | | | |
| --- | --- | --- | --- | --- | --- | --- | --- |
|  | Retail | Grocery | Parks | Transit | Workplace | Residential |  |
| Community of Madrid | -61 | -31 | -47 | -56 | -50 | 22 |  |
| Barcelona Urban Area | -59 | -30 | -46 | -52 | -45 | 20 |  |
| Île-de-France | -57 | -23 | -53 | -53 | -48 | 22 |  |
| New York-Newark-Jersey City | -24 | -2 | 16 | -30 | -28 | 12 |  |
| Greater London | -50 | -18 | -12 | -49 | -44 | 18 |  |
| Hong Kong | -24 | -4 | -14 | -31 | -18 | 14 |  |
| Mexico City Metropolitan Area | -37 | -15 | -33 | -35 | -29 | 14 |  |

Note: Negative values correspond to decreases in mobility, while positive numbers correspond to increases.

**Table S2.** Description of monitoring sites used to calculate net changes in air pollutant concentrations during the COVID-19 lockdown within the MCMA.

| Site | Environment | Code | Geographical setting within the MCMA | Location relative to major emission sources |  |
| --- | --- | --- | --- | --- | --- |
| Miguel Hidalgo | Traffic | TRA | Centre-West | Downwind of a high load motorway | |
| Tlalnepantla | Industrial | IND | Centre-North | Downwind of industrial sources | |
| Merced | Commercial | COM | Centre | Close to a major food market | |
| Cuautitlán | Urban background upwind | UBN | North | Upwind urban emissions | |
| Pedregal | Residential | RES | Centre-South | Downwind most of the city emission sources | |
| Ajusco medio | Urban background downwind | UBS | South | Close to a forest | |

**Table S3.** Residual standard error expressed in percentage and F-statistic for the air pollutants time-series modelling using Fourier series within the MCMA.

| Pollutant | CO | | NO_2_ | | O_3_ | | PM_10_ | | PM_2.5_ | | SO_2_ | |
| --- | --- | --- | --- | --- | --- | --- | --- | --- | --- | --- | --- | --- |
| Site | Error | F-Stat | Error | F-Stat | Error | F-Stat | Error | F-Stat | Error | F-Stat | Error | F-Stat |
| COM | 0.2 | 2E-266 | 7.9 | 4E-97 | 7.9 | 3E-171 | 16.6 | 4E-130 | 9.9 | 3E-74 | 5.5 | 4E-55 |
| IND | 0.2 | 1E-235 | 7.9 | 3E-94 | 7.4 | 4E-134 | 15.0 | 3E-112 | 8.8 | 2E-55 | 7.1 | 4E-39 |
| RES | 0.2 | 1E-297 | 5.8 | 4E-128 | 10.3 | 1E-133 | 13.2 | 1E-147 | 9.2 | 1E-83 | 5.0 | 3E-44 |
| TRA | 0.2 | 8E-218 | 8.5 | 7E-76 | 8.5 | 2E-125 | 13.2 | 2E-161 | 9.6 | 5E-81 | 4.5 | 6E-52 |
| UBN | 0.2 | 1E-185 | 5.4 | 1E-155 | 7.0 | 2E-136 | 16.5 | 2E-201 | 9.4 | 5E-71 | 6.7 | 2E-21 |
| UBS | 0.1 | 3E-300 | 5.2 | 2E-88 | 11.5 | 1E-108 | 16.0 | 4E-142 | 8.2 | 7E-48 | 3.3 | 2E-77 |

**Table S4.** Main statistics for hourly anomalies during 2016–2019 and Phases 2–3 of the COVID-19 lockdown within the MCMA at the selected monitoring sites.

| Pollutant | Site | Phase 2 | | | | | | | |  | Phase 3 | | | | | | | |
| --- | --- | --- | --- | --- | --- | --- | --- | --- | --- | --- | --- | --- | --- | --- | --- | --- | --- | --- |
|  |  | 2016-2019 | | | | Lockdown | | | |  | 2016-2019 | | | | Lockdown | | | |
|  |  | Min | Avg | SD | Max | Min | Avg | SD | Max |  | Min | Avg | SD | Max | Min | Avg | SD | Max |
| CO | UBN | -0.55 | -0.13 | 0.35 | 1.98 | -0.36 | -0.05 | 0.26 | 1.34 |  | -0.70 | -0.12 | 0.35 | 2.29 | -0.32 | -0.05 | 0.20 | 1.09 |
| (ppm) | IND | -0.64 | -0.01 | 0.46 | 2.85 | -0.30 | 0.04 | 0.21 | 0.90 |  | -0.63 | 0.03 | 0.43 | 2.65 | -0.28 | -0.01 | 0.20 | 0.73 |
|  | COM | -0.68 | 0.02 | 0.48 | 3.27 | -0.27 | 0.10 | 0.28 | 1.61 |  | -0.59 | 0.06 | 0.48 | 3.51 | -0.29 | 0.05 | 0.25 | 1.51 |
|  | RES | -0.91 | -0.34 | 0.30 | 1.18 | -0.30 | -0.06 | 0.13 | 0.45 |  | -0.78 | -0.21 | 0.28 | 1.40 | -0.26 | -0.05 | 0.11 | 0.36 |
|  | TRA | -0.67 | 0.06 | 0.42 | 2.24 | -0.25 | 0.03 | 0.20 | 1.32 |  | -0.56 | 0.03 | 0.36 | 1.95 | -0.24 | 0.02 | 0.17 | 0.77 |
|  | UBS | -0.42 | -0.04 | 0.19 | 0.98 | -0.13 | 0.06 | 0.11 | 0.38 |  | -0.34 | 0.06 | 0.24 | 1.35 | -0.16 | 0.03 | 0.10 | 0.36 |
| NO_2_ | UBN | -19 | 0 | 12 | 44 | -17 | -3 | 9 | 33 |  | -20 | 1 | 12 | 58 | -16 | -3 | 9 | 26 |
| (ppb) | IND | -28 | -1 | 15 | 64 | -25 | -5 | 11 | 31 |  | -25 | 2 | 14 | 66 | -22 | -6 | 10 | 21 |
|  | COM | -28 | -1 | 15 | 71 | -27 | -5 | 12 | 29 |  | -25 | 2 | 15 | 75 | -26 | -7 | 11 | 34 |
|  | RES | -19 | 0 | 11 | 36 | -16 | -5 | 7 | 20 |  | -19 | 2 | 12 | 61 | -17 | -7 | 6 | 17 |
|  | TRA | -26 | 1 | 14 | 54 | -24 | -7 | 10 | 20 |  | -22 | 2 | 14 | 112 | -22 | -8 | 9 | 21 |
|  | UBS | -15 | -1 | 9 | 39 | -10 | 0 | 6 | 20 |  | -14 | 0 | 9 | 44 | -10 | -2 | 5 | 15 |
| O_3_ | UBN | -34 | -3 | 26 | 102 | -35 | -4 | 25 | 66 |  | -35 | 1 | 29 | 92 | -36 | -2 | 26 | 70 |
| (ppb) | IND | -32 | -1 | 26 | 93 | -33 | 3 | 25 | 67 |  | -33 | 2 | 28 | 109 | -34 | 2 | 22 | 69 |
|  | COM | -35 | -4 | 27 | 105 | -38 | 3 | 32 | 107 |  | -36 | 2 | 31 | 122 | -38 | 5 | 29 | 77 |
|  | RES | -46 | -1 | 31 | 113 | -47 | -2 | 28 | 78 |  | -45 | 2 | 35 | 133 | -49 | -1 | 25 | 71 |
|  | TRA | -36 | -3 | 28 | 109 | -36 | 8 | 31 | 96 |  | -36 | 3 | 33 | 154 | -36 | 7 | 28 | 83 |
|  | UBS | -47 | -1 | 24 | 103 | -33 | 10 | 24 | 80 |  | -46 | 4 | 28 | 119 | -31 | 11 | 21 | 78 |
| PM_10_ | UBN | -61 | 2 | 38 | 240 | -48 | 1 | 40 | 257 |  | -54 | 5 | 35 | 329 | -44 | 3 | 36 | 217 |
| (µg m^-3^) | IND | -57 | 1 | 29 | 138 | -42 | -7 | 17 | 61 |  | -48 | 4 | 26 | 156 | -50 | -12 | 19 | 171 |
|  | COM | -56 | 2 | 27 | 124 | -45 | -1 | 22 | 149 |  | -46 | 7 | 25 | 122 | -49 | -11 | 19 | 149 |
|  | RES | -35 | 14 | 27 | 130 | -37 | -4 | 14 | 38 |  | -36 | 18 | 25 | 157 | -44 | -16 | 13 | 48 |
|  | TRA | -40 | 0 | 21 | 131 | -36 | -3 | 14 | 64 |  | -37 | 3 | 19 | 180 | -29 | -5 | 14 | 64 |
|  | UBS | -52 | -15 | 20 | 116 | -58 | -25 | 17 | 27 |  | -46 | -9 | 19 | 134 | -54 | -30 | 12 | 50 |
| PM_2.5_ | UBN | -26 | 2 | 17 | 116 | -25 | -2 | 12 | 34 |  | -26 | 4 | 14 | 64 | -26 | -5 | 12 | 36 |
| (µg m^-3^) | IND | -27 | -1 | 15 | 75 | -24 | -1 | 11 | 35 |  | -28 | 3 | 14 | 61 | -25 | -5 | 11 | 27 |
|  | COM | -28 | 0 | 15 | 56 | -20 | 2 | 10 | 32 |  | -29 | 5 | 16 | 70 | -25 | -3 | 11 | 31 |
|  | RES | -22 | 4 | 14 | 65 | -25 | -4 | 9 | 22 |  | -23 | 8 | 15 | 133 | -25 | -7 | 9 | 18 |
|  | TRA | -25 | -1 | 13 | 53 | -19 | 1 | 9 | 26 |  | -24 | 3 | 13 | 63 | -21 | -2 | 9 | 22 |
|  | UBS | -21 | -1 | 12 | 88 | -17 | 1 | 10 | 28 |  | -20 | 3 | 13 | 81 | -19 | -5 | 7 | 15 |
| SO_2_ | UBN | -6 | 1 | 14 | 187 | -5 | -2 | 5 | 43 |  | -5 | 0 | 9 | 89 | -4 | -2 | 3 | 19 |
| (ppb) | IND | -7 | 1 | 14 | 125 | -5 | -2 | 3 | 24 |  | -6 | 0 | 10 | 162 | -4 | -1 | 4 | 53 |
|  | COM | -5 | 1 | 10 | 92 | -3 | -1 | 2 | 18 |  | -4 | 0 | 7 | 136 | -3 | -1 | 2 | 23 |
|  | RES | -5 | -1 | 4 | 35 | -3 | -1 | 1 | 5 |  | -4 | -1 | 4 | 49 | -2 | -2 | 2 | 15 |
|  | TRA | -4 | 0 | 7 | 74 | -4 | -2 | 1 | 5 |  | -3 | 0 | 6 | 85 | -3 | -2 | 3 | 32 |
|  | UBS | -3 | 0 | 4 | 30 | -2 | -1 | 1 | 4 |  | -2 | 0 | 3 | 42 | -2 | -1 | 2 | 13 |

**Table S5.** Significant correlations at *p* < 0.05 between air pollutant concentrations and mobility within the MCMA during COVID-19 lockdown Phases 2–3 at the selected monitoring sites.

| Pollutant | Mobility category | Monitoring site | | | | | |
| --- | --- | --- | --- | --- | --- | --- | --- |
|  |  | UBN | IND | COM | RES | TRA | UBS |
| CO | Retail | 0.42 | - | - | - | 0.66 | - |
|  | Grocery | - | - | - | - | 0.63 | - |
|  | Parks | 0.53 | - | 0.41 | 0.38 | 0.73 | - |
|  | Transit | 0.41 | - | - | - | 0.61 | - |
|  | Workplace | - | - | - | - | - | - |
|  | Residential | - | - | - | - | - | - |
| NO_2_ | Retail | 0.40 | 0.57 | 0.69 | 0.52 | 0.59 | 0.66 |
|  | Grocery | 0.43 | 0.60 | 0.57 | 0.50 | 0.55 | 0.60 |
|  | Parks | 0.56 | 0.77 | 0.79 | 0.63 | 0.74 | 0.75 |
|  | Transit | - | - | 0.47 | 0.45 | 0.45 | 0.52 |
|  | Workplace | - | - | - | - | - | - |
|  | Residential | - | - | - | - | - | - |
| O_3_ | Retail | - 0.64 | - 0.46 | - 0.45 | - | - | - |
|  | Grocery | - 0.42 | - | - 0.46 | - | - | - |
|  | Parks | - 0.65 | - 0.55 | - 0.56 | - | - 0.37 | - |
|  | Transit | - 0.55 | - 0.39 | - 0.43 | - | - | - |
|  | Workplace | - | - | - | - | - | - |
|  | Residential | - | - | - | - | - | - |
| PM_10_ | Retail | 0.52 | - | 0.55 | - | - | 0.48 |
|  | Grocery | - | - | - | - | - | - |
|  | Parks | 0.46 | - | - | - | - | 0.37 |
|  | Transit | - | - | - | - | - | - |
|  | Workplace | - | - | - | - | - | - |
|  | Residential | - | - | - | - | - | - |
| PM_2.5_ | Retail | - | - | - | - | - | - 0.84 |
|  | Grocery | - | - | - | - | - | - 0.81 |
|  | Parks | - | - | - | - | - | - 0.84 |
|  | Transit | - | - | - | - | - | - 0.74 |
|  | Workplace | - | - | - | - | - | - |
|  | Residential | - | - | - | - | - | - |
| SO_2_ | Retail | - | 0.39 | - | 0.39 | 0.40 | 0.37 |
|  | Grocery | - | - | - | - | - | - |
|  | Parks | - | - | - | - | - | - |
|  | Transit | - | - | - | - | - | - |
|  | Workplace | - 0.37 | - | - | - | - | - |
|  | Residential | - | - | - | - | - | - |

**Table S6.** Significant correlations at *p* < 0.05 between air pollutant concentrations and mobility within the MCMA during COVID-19 lockdown Phases 2–3 at the selected monitoring sites.

| Pollutant | Lockdown phase | Metric | Monitoring site | | | | | |
| --- | --- | --- | --- | --- | --- | --- | --- | --- |
|  |  |  | UBN | IND | COM | RES | TRA | UBS |
| CO | Phase 2 | 4-yr baseline | -0.034 | 0.050 | 0.067 | 0.049 | -0.030 | 0.101 |
|  | Phase 2 | Year-by-year | -0.031 | 0.047 | 0.073 | 0.053 | -0.032 | 0.101 |
|  | Phase 3 | Overall period | -0.063 | -0.034 | -0.008 | 0.000 | -0.003 | -0.034 |
|  | Phase 3 | Year-by-year | -0.066 | -0.031 | -0.007 | 0.000 | -0.003 | -0.034 |
|  | Phase 2 | Difference in ppm | 0.003 | 0.003 | 0.006 | 0.004 | 0.002 | 0.001 |
|  | Phase 3 | Difference in ppm | 0.003 | 0.003 | 0.001 | 0.000 | 0.000 | 0.000 |
|  | Phase 2 | Difference in % | 9.23 | 6.45 | 8.44 | 8.31 | 7.23 | 0.50 |
|  | Phase 3 | Difference in % | 4.69 | 8.59 | 9.84 | 0.00 | 7.52 | 1.48 |
| NO_2_ | Phase 2 | 4-yr baseline | -3.45 | -3.70 | -4.00 | -4.93 | -7.57 | 0.20 |
|  | Phase 2 | Year-by-year | -3.79 | -3.40 | -4.48 | -5.23 | -7.69 | 0.19 |
|  | Phase 3 | Overall period | -3.92 | -8.00 | -9.86 | -9.03 | -10.32 | -1.98 |
|  | Phase 3 | Year-by-year | -3.81 | -8.01 | -9.90 | -8.94 | -10.03 | -1.90 |
|  | Phase 2 | Difference in ppm | 0.35 | 0.30 | 0.48 | 0.30 | 0.11 | 0.01 |
|  | Phase 3 | Difference in ppm | 0.11 | 0.01 | 0.04 | 0.10 | 0.29 | 0.08 |
|  | Phase 2 | Difference in % | 9.55 | 8.45 | 10.28 | 5.89 | 1.49 | 6.19 |
|  | Phase 3 | Difference in % | 2.94 | 0.12 | 0.39 | 0.00 | 2.83 | 4.08 |
| O_3_ | Phase 2 | 4-yr baseline | -0.04 | 4.46 | 6.91 | -0.95 | 10.98 | 11.22 |
|  | Phase 2 | Year-by-year | -0.43 | 4.10 | 6.65 | -1.02 | 9.97 | 11.34 |
|  | Phase 3 | Overall period | -3.99 | -0.06 | 2.70 | -3.53 | 4.26 | 7.36 |
|  | Phase 3 | Year-by-year | -4.16 | -0.07 | 2.51 | -3.86 | 3.99 | 7.15 |
|  | Phase 2 | Difference in ppm | 0.39 | 0.36 | 0.26 | 0.07 | 1.01 | 0.13 |
|  | Phase 3 | Difference in ppm | 0.17 | 0.01 | 0.19 | 0.33 | 0.27 | 0.21 |
|  | Phase 2 | Difference in % | 167.33 | 8.43 | 3.83 | 7.08 | 9.68 | 1.11 |
|  | Phase 3 | Difference in % | 4.18 | 9.15 | 7.26 | 0.00 | 6.67 | 2.91 |
| SO_2_ | Phase 2 | 4-yr baseline | -2.60 | -2.53 | -2.02 | -0.88 | -2.20 | -0.88 |
|  | Phase 2 | Year-by-year | -2.63 | -2.42 | -2.20 | -0.80 | -2.22 | -0.80 |
|  | Phase 3 | Overall period | -1.77 | -0.87 | -1.07 | -0.16 | -1.84 | -0.69 |
|  | Phase 3 | Year-by-year | -1.88 | -0.83 | -0.99 | -0.16 | -1.92 | -0.68 |
|  | Phase 2 | Difference in ppm | 0.03 | 0.12 | 0.19 | 0.09 | 0.02 | 0.08 |
|  | Phase 3 | Difference in ppm | 0.11 | 0.04 | 0.08 | 0.00 | 0.08 | 0.01 |
|  | Phase 2 | Difference in % | 1.00 | 4.73 | 8.78 | 10.29 | 0.79 | 9.69 |
|  | Phase 3 | Difference in % | 5.77 | 4.82 | 7.77 | 0.00 | 4.15 | 1.98 |
| PM_10_ | Phase 2 | 4-yr baseline | -0.08 | -7.98 | -3.59 | -5.41 | -1.04 | -9.93 |
|  | Phase 2 | Year-by-year | -0.11 | -8.79 | -3.84 | -5.92 | -1.15 | -10.75 |
|  | Phase 3 | Overall period | -1.23 | -16.40 | -17.92 | -18.20 | -9.63 | -16.84 |
|  | Phase 3 | Year-by-year | -1.17 | -14.92 | -16.29 | -20.55 | -10.59 | -18.32 |
|  | Phase 2 | Difference in ppm | 0.03 | 0.81 | 0.25 | 0.51 | 0.10 | 0.82 |
|  | Phase 3 | Difference in ppm | 0.06 | 1.48 | 1.63 | 2.35 | 0.96 | 1.48 |
|  | Phase 2 | Difference in % | 32.24 | 9.65 | 6.83 | 9.03 | 9.54 | 7.91 |
|  | Phase 3 | Difference in % | 4.92 | 9.46 | 9.52 | 0.00 | 9.47 | 8.40 |
| PM_2.5_ | Phase 2 | 4-yr baseline | -3.67 | -0.78 | 2.14 | -0.80 | 1.94 | 3.00 |
|  | Phase 2 | Year-by-year | -5.68 | -0.86 | 1.95 | -0.89 | 1.76 | 2.75 |
|  | Phase 3 | Overall period | -8.51 | -7.94 | -8.04 | -8.08 | -6.59 | -8.55 |
|  | Phase 3 | Year-by-year | -9.00 | -8.29 | -7.38 | -8.83 | -7.16 | -9.45 |
|  | Phase 2 | Difference in ppm | 2.00 | 0.08 | 0.19 | 0.09 | 0.18 | 0.25 |
|  | Phase 3 | Difference in ppm | 0.49 | 0.35 | 0.66 | 0.75 | 0.56 | 0.90 |
|  | Phase 2 | Difference in % | 42.83 | 9.80 | 9.22 | 10.15 | 9.73 | 8.63 |
|  | Phase 3 | Difference in % | 5.65 | 4.36 | 8.57 | 0.00 | 8.19 | 10.02 |

## Supplementary Figures

##
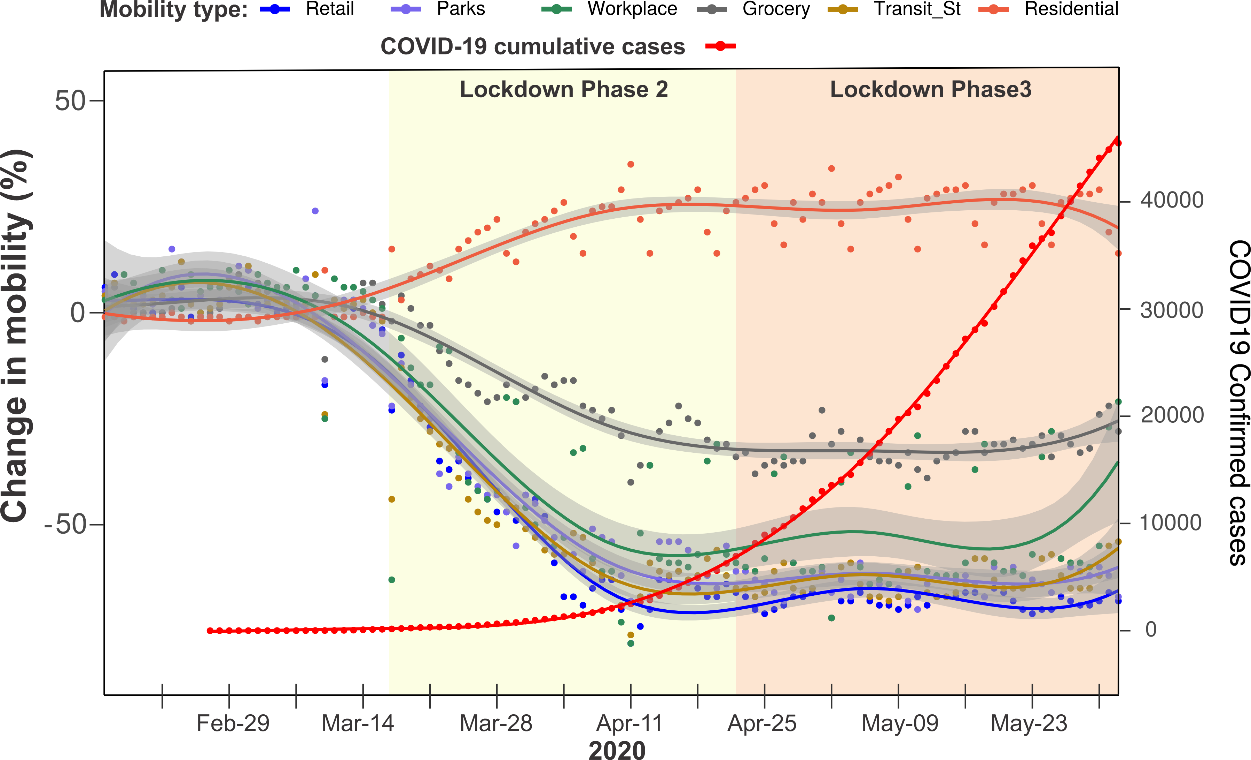


## Figure S1. Daily confirmed COVID-19 cases and changes in urban mobility during the COVID-19 lockdown Phases 2–3 within the MCMA. The solid lines show the smoothing of the mobility trends.

##
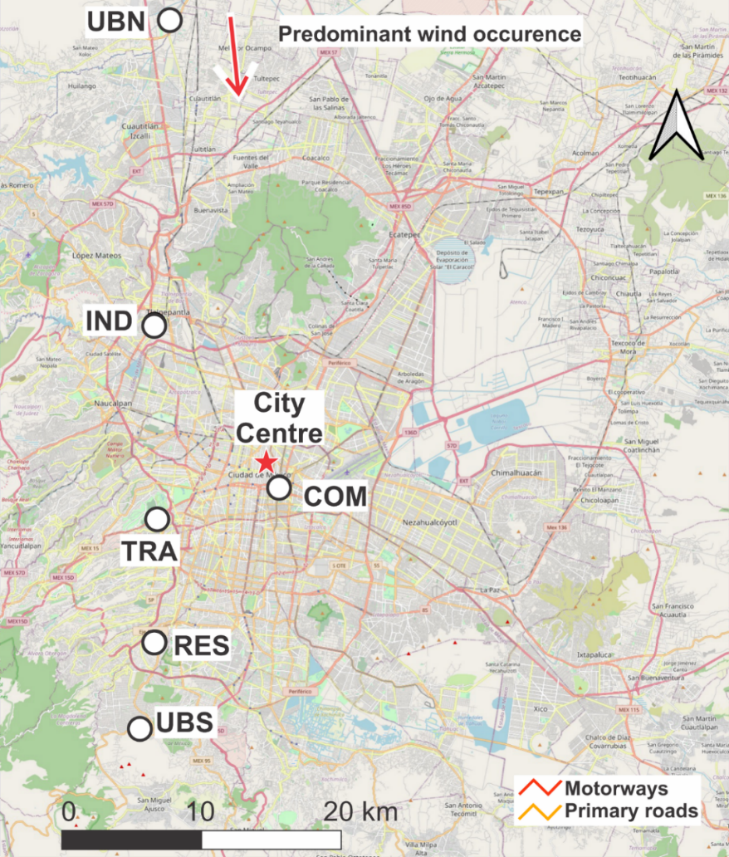


## Figure S2. Location of the monitoring sites within the MCMA used in this study to calculate changes in air pollutant concentrations during the COVID-19 lockdown.


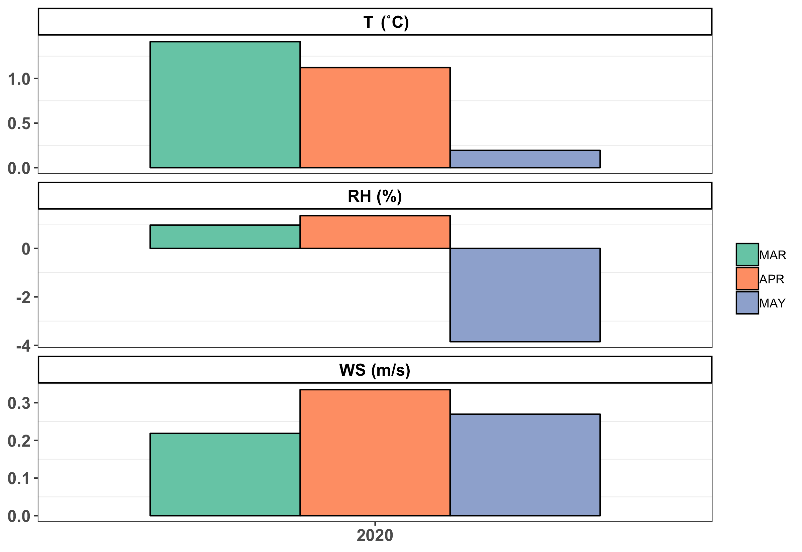


**Figure S3.** Monthly anomalies in surface temperature (TMP.), relative humidity (RH) and wind speed (WS) for the COVID-19 lockdown period (March, April and May 2020) within the MCMA.


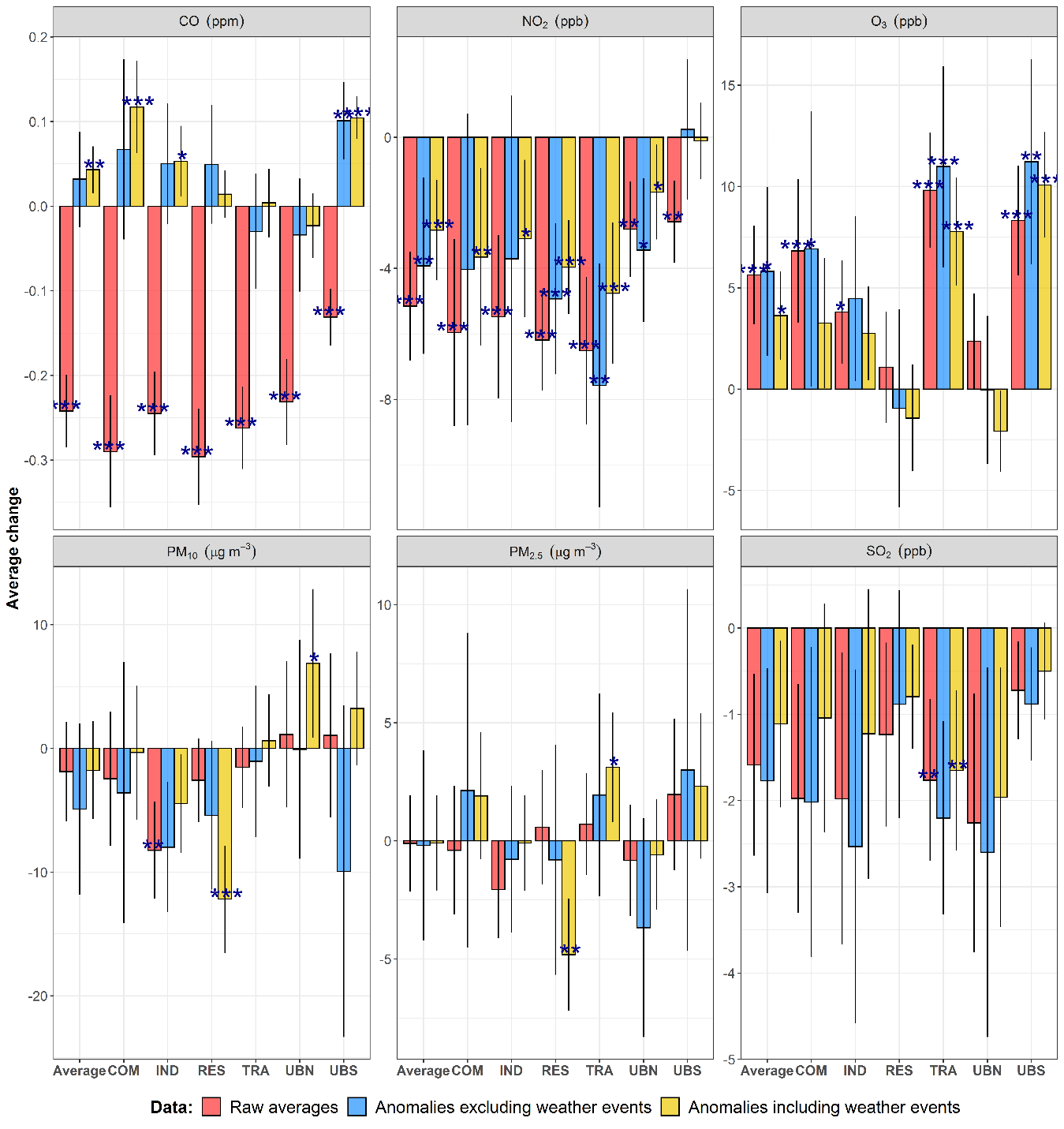


**Figure S4.** Comparison of air pollutant changes during the Phase 2 calculated using raw observations, anomalies excluding stagnant conditions and rain events, and without weather events. The vertical lines show the 95 % confidence intervals.


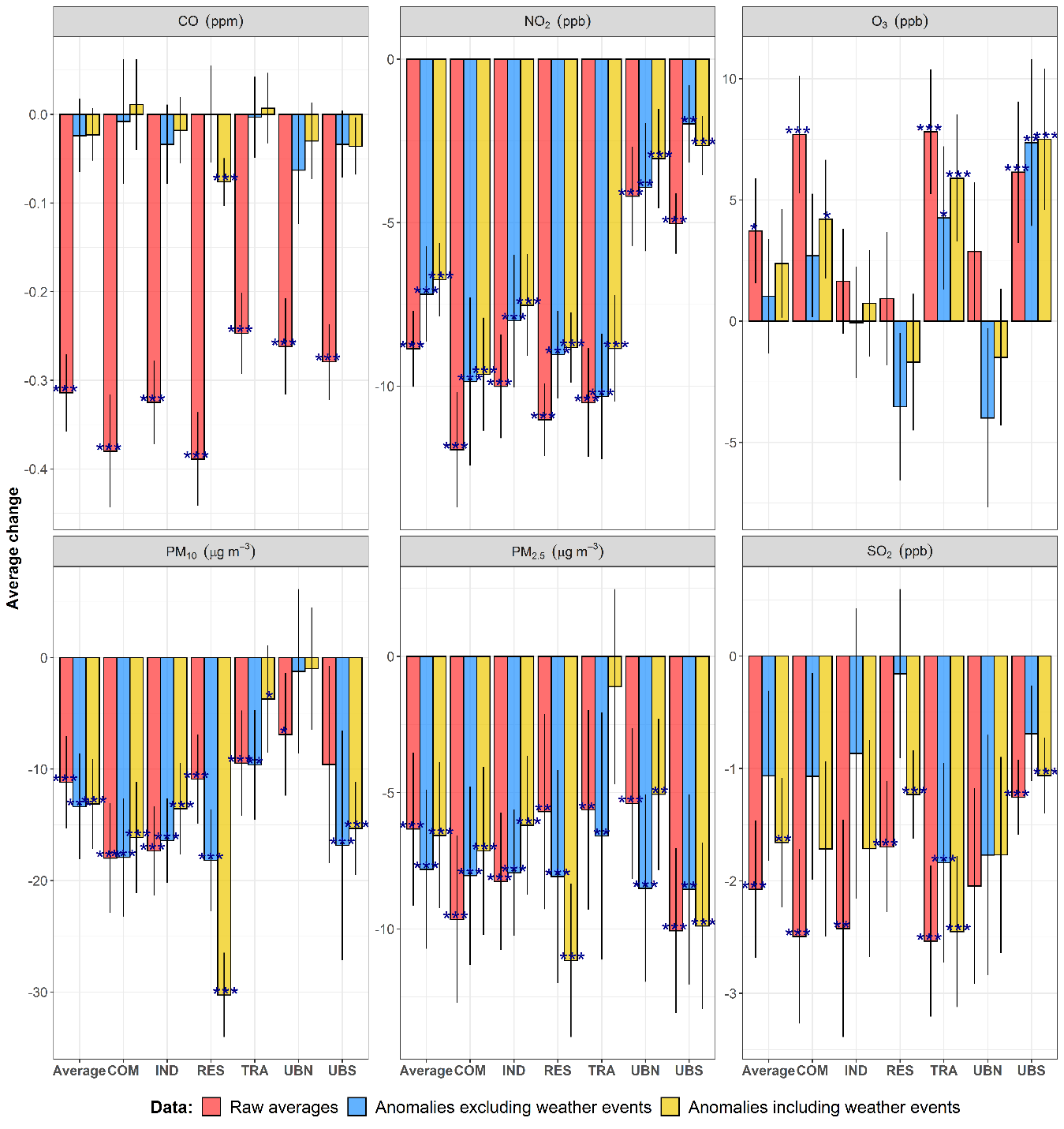


**Figure S5.** Comparison of air pollutant changes during the Phase 3 calculated using raw observations, anomalies excluding stagnant conditions and rain events, and without weather events. The vertical lines show the 95 % confidence intervals.


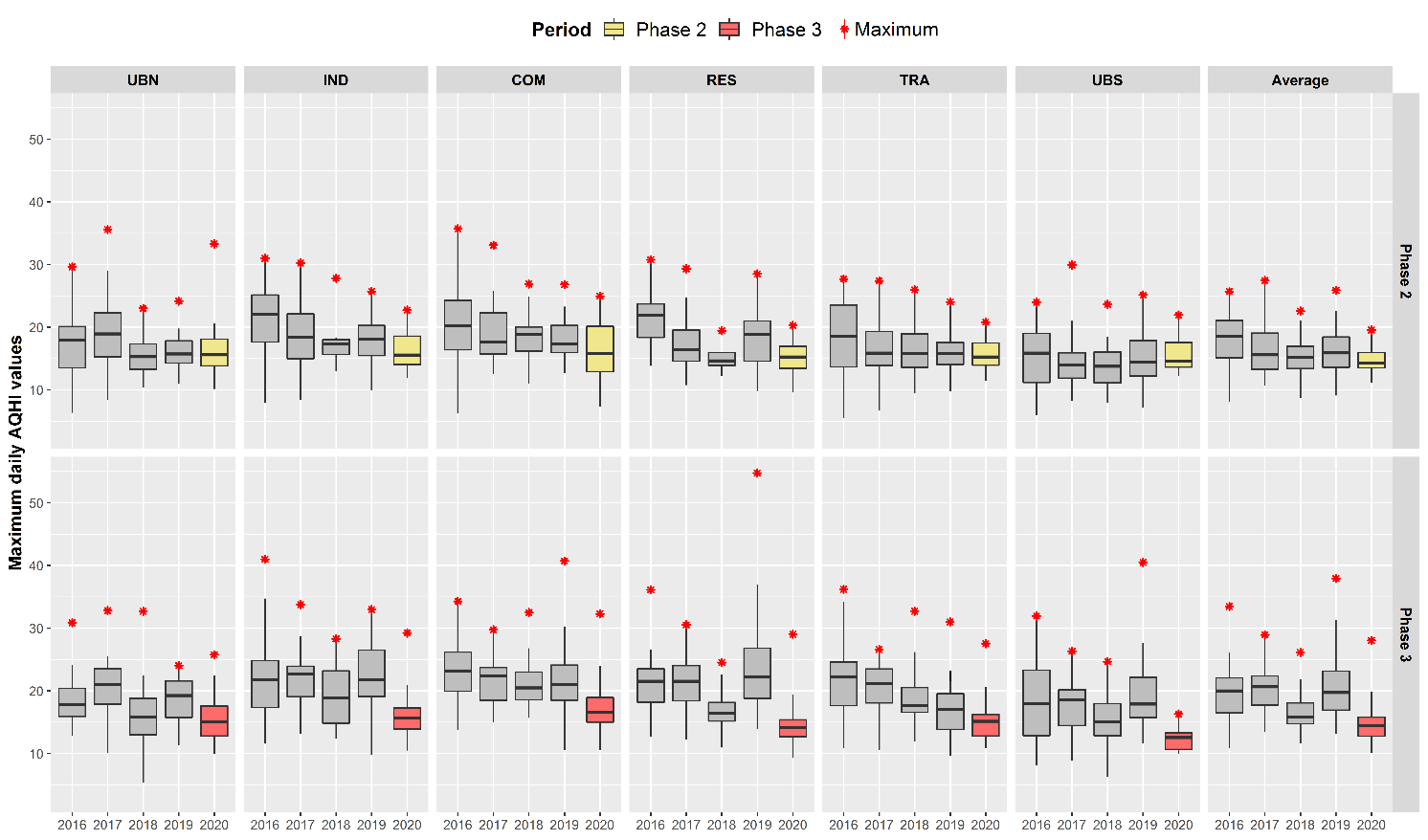


**Figure S6.** Boxplot of maximum daily AQHI values at each monitoring site within the MCMA during 2016-2019 (baseline) and COVID-19 lockdown Phases 2–3. The red star shows maximum daily AQHI values during each period.

**
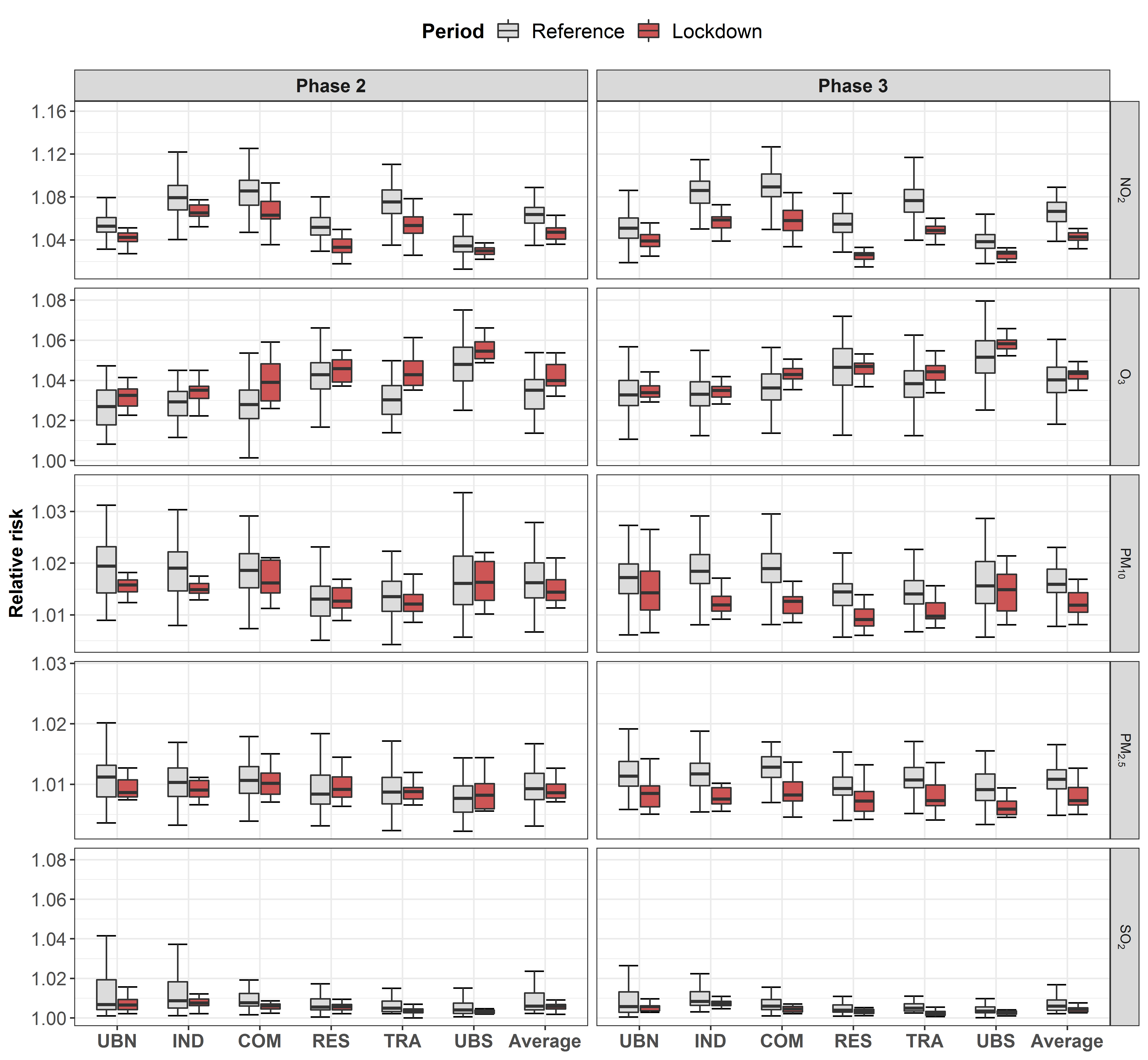
**

**Figure S7.** Boxplot of relative risk used to calculate the excess risk for the criteria air pollutant during the reference (2016–2019) and COVID-19 lockdown periods within the MCMA. No relative risk was calculated for CO as its concentration did not exceed the limit of 2 mg m^-3^ considered as safe.


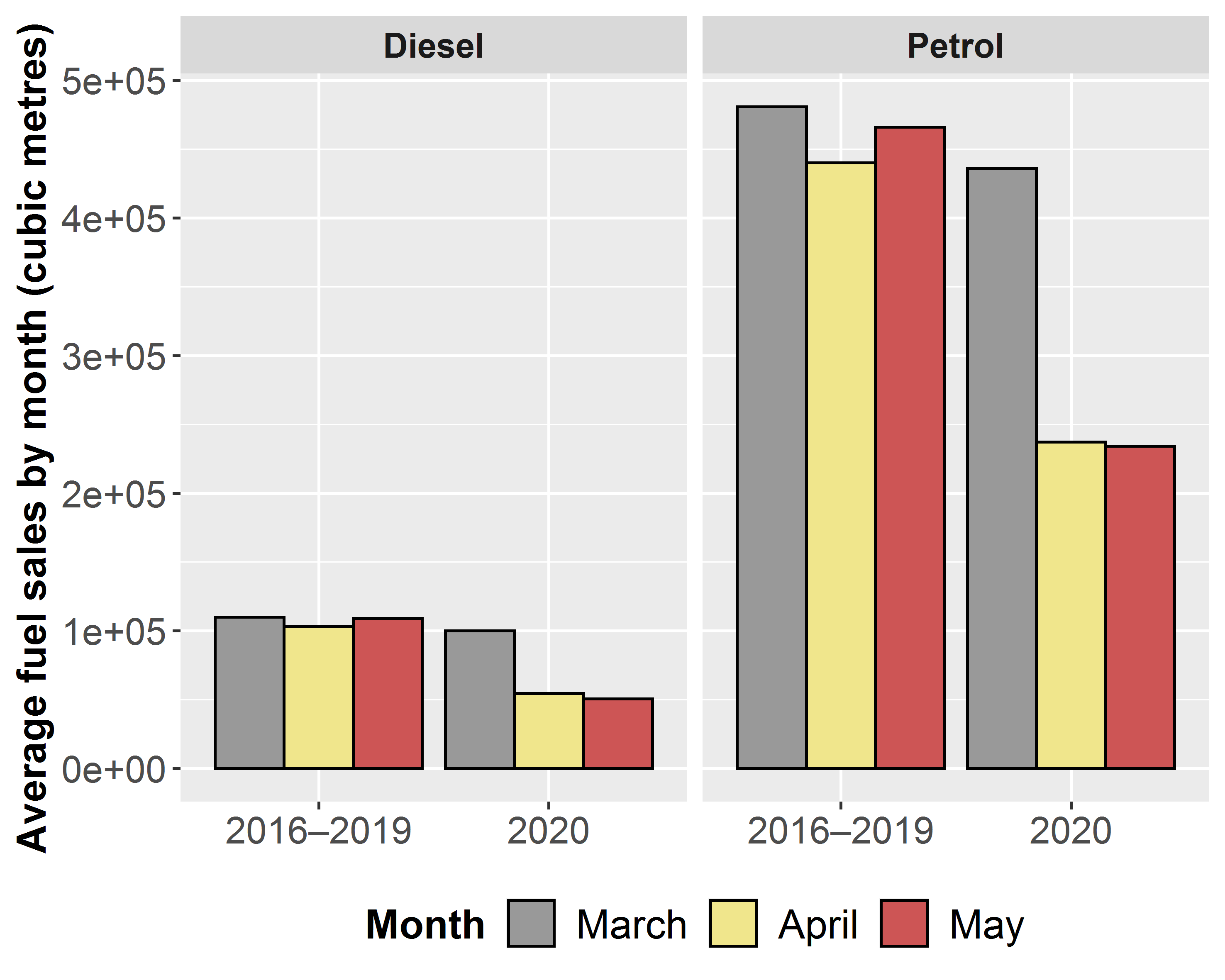


**Figure S8.** Average petrol and diesel monthly sales within the MCMA from March to May which include the lockdown Phases 2–3 and the corresponding periods from 2016 to 2019.
